# RNA-binding proteins connect Exon usage to the chromatin

**DOI:** 10.1093/nargab/lqaf161

**Published:** 2025-12-10

**Authors:** Hanah Robertson, Hoang T T Do, Volkhard Helms

**Affiliations:** Center for Bioinformatics, Saarland University, 66041 Saarbrücken, Germany; Center for Bioinformatics, Saarland University, 66041 Saarbrücken, Germany; Center for Bioinformatics, Saarland University, 66041 Saarbrücken, Germany

## Abstract

Exonic enrichment of histone marks hints at their role in regulating alternative splicing. This study aims to connect the transcriptome and epigenome in the context of splicing outcomes in embryonic cell lines. The tools rMATS and MANorm were used to obtain estimates of differential inclusion of exons and differential enrichment of epigenetic signals, respectively. Two classes of alternative exons were identified in embryonic cell lines: those differentially co-occurring with at least one mark among H3K27ac, H3K27me3, H3K36me3, H3K9me3, and H3K4me3, and those marked by neither of these marks. Binary classifiers were trained using RNA-binding protein (RBP) binding affinities on the flanking regions of these exons. This resulted in a set of RBPs, whose putative binding was predicted to associate local chromatin modification marking an exon with its differential inclusion, some of which have been experimentally shown to interact with histone mark reader proteins. We speculate that sequence signals harbored at exon-intron flanks regulate differential splicing of exons, marked by at least one of the five epigenetic signatures. Finally, eCLIP data from ENCODE for the HepG2 and K562 cell lines support TIA1 and U2AF2 as potential episplicing RBPs, as predicted by our model in the embryonic cell lines.

## Introduction

An important aspect of protein diversity is the alternative inclusion of exons in mRNA transcripts of genes. It is ubiquitously known that by regulating chromatin accessibility, histone modifications promote or repress transcription [1]. Splicing events that occur in a co-transcriptional manner suggest that the regulatory effects of the chromatin state may extend beyond DNA to the transcribed RNA, influencing splicing events. Multiple studies have shown that specific post-transcriptional histone modifications may be enriched at exons [2–5]. Hence, the chromatin context is speculated to regulate splicing, either indirectly by guiding transcription kinetics [6–9], or mechanistically through chromatin-adaptor complexes [10–12].

In addition to studies that uncovered chromatin-associated splicing events at gene resolution [10, 13, 14], machine learning approaches have been used to decipher relationships between epigenetic modifications and splicing events at a larger scale. One study on splicing events in the mammalian brain used epigenomic signals to predict the type of alternative splicing event of exons through a Random forest classifier [15]. Deep-learning models have been used to predict inclusion levels of exons based on the epigenetic signals occurring in their neighborhood [16, 17]. A Random Forest model was used by Agirre et al. to classify exons into four different categories of inclusion levels, based on the state of the adjacent chromatin [18]. In a recent preprint, Manz et al. employ Random Forest models and find observed and imputed epigenetic features predictive of exon usage status [19].

**Figure 1.**
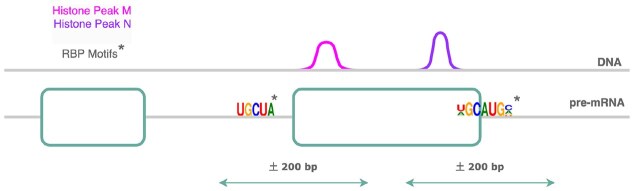
DHM signals and RNA–RBP interaction events were studied at the exon-intron margins of alternative exons.

Following up on previous studies that reported that differential exon usage (DEU) is associated with local epigenetic marks in embryonic development [20–22], this study focused on the analysis of epigenetics-associated splicing events in early differentiation. The usage of an exon is determined by the coordination of various cis and trans-regulatory elements, the most fundamental of which is the recognition of the splice site by members of the spliceosome. Here, the putative binding affinities of RNA-binding proteins (RBP) at the exon-intron junctions of differentially used exons were used to predict their local chromatin state (Fig. 1). Via a Random Forest classifier, a set of RBPs was determined that were purported to show either stronger or weaker binding to the flanking regions of exons marked by a differential histone modification (DHM), relative to those of alternative exons not marked by either of the histone marks of interest. Upon examining the motifs of these RBPs, histone mark-specific enrichment of sequence signals was found, suggesting the presence of another layer of splicing regulation connecting the chromatin to the transcriptome. In fact, for the HepG2 and K562 cell lines, there exists eCLIP evidence from the ENCODE project supporting the binding of two of these predicted RBPs, TIA1, and U2AF2, in parallel to changes in the H3K36me3 signal adjacent to skipped exons.

## Materials and methods

### High-throughput sequencing datasets

Transcriptomic data were obtained from the resources of the ENCODE project for ectodermal, endodermal, mesodermal, neuronal stem cells, H1, K562, and HepG2 cell lines, respectively. The accompanying ChIP-Seq data for these biosamples were downloaded for histone marks H3K27ac, H3K27me3, H3K4me3, H3K9me3, and H3K36me3. The eCLIP data available for HepG2 and K562 cell lines were obtained from the ENCODE3 project for 47 proteins. A detailed list of all considered data is shown in Supplementary Table S1.

### Candidate Exons

**Figure 2.**
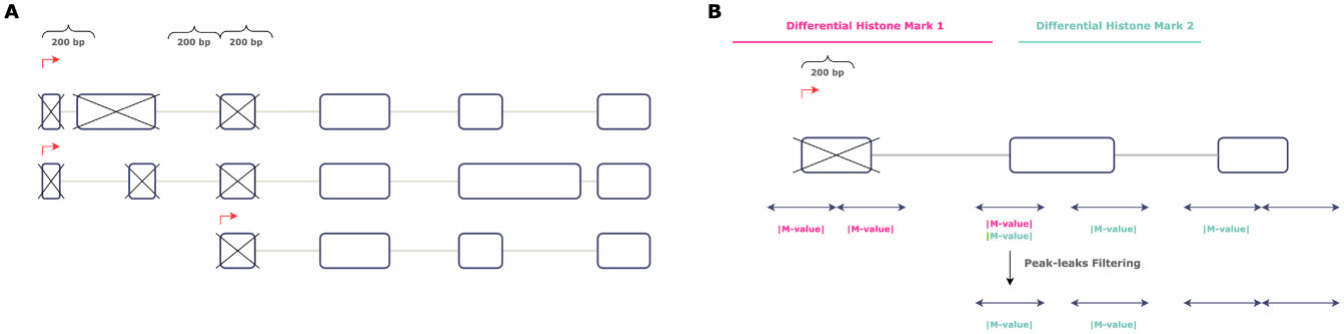
**(A)** Exons occurring in a window of $\pm {200bp}$ of any TSS were excluded. **(B)** Peaks annotating flanks of exons within 200 bp of TSS were not used in this analysis. Peaks were required to cover at least half of an exon in order to be annotated to its flanks.

All annotations were based on the Gencode human reference GRCh38, release V24. To focus on splicing events from well-supported transcripts, only exons belonging to transcripts with support levels (TSL) 1–3 were considered. Of these exons, those found within a 200 bp window of any annotated transcription start sites (TSS) with support levels 1–5 were excluded in order to avoid studying the effects of chromatin marks associated with transcription initiation (Fig. 2A). The remaining exons were considered candidate exons of interest. The exon coordinates were obtained from the GFF3 file. A combination of BEDtools [23] and bash utilities was used to retrieve the candidate exons and their flanking sequences ($\pm$200 bp).

### DEU analysis

**Figure 3.**
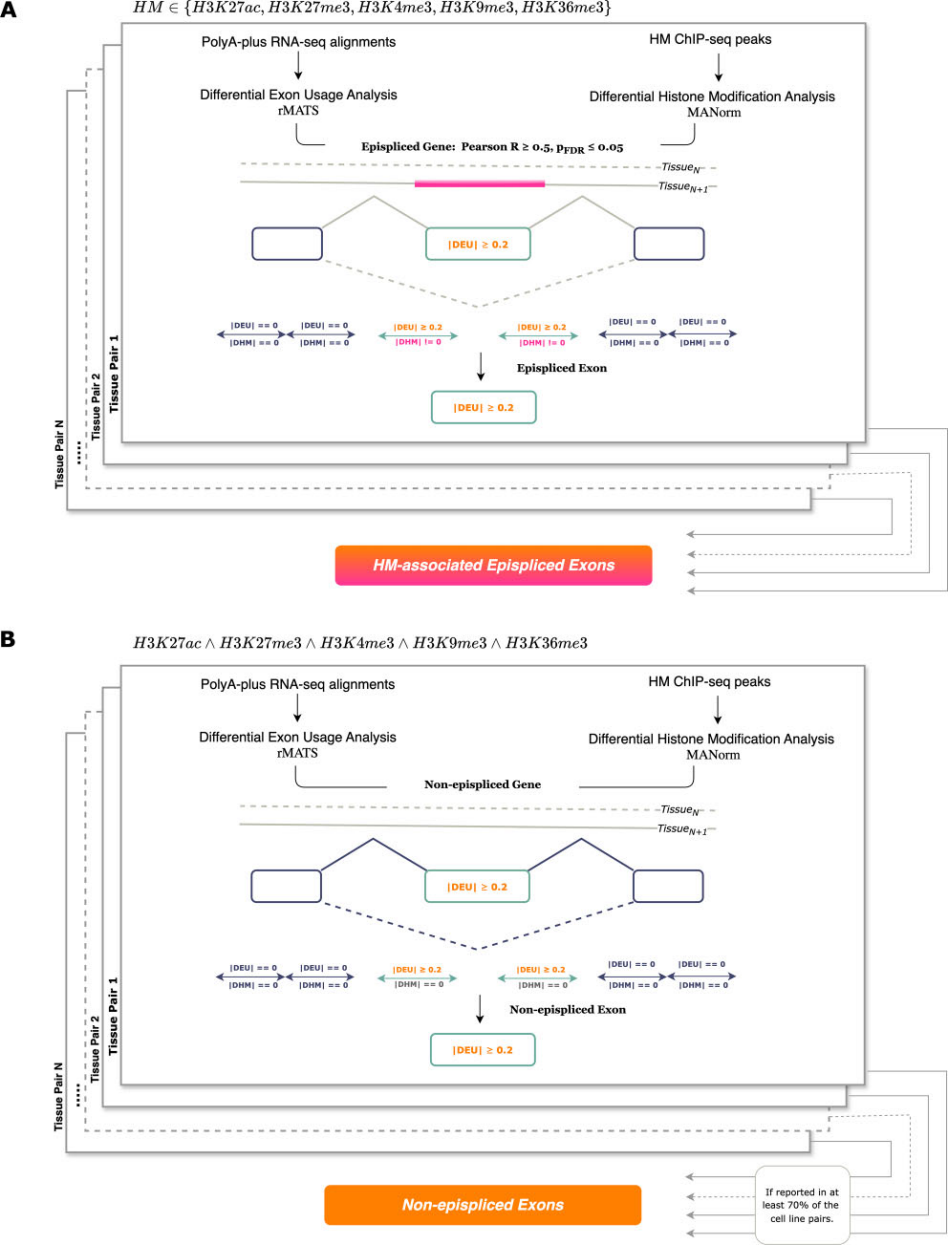
**(A)** By correlating DEU and DHM scores annotated to exon flanks in a gene-wise manner, genes with an absolute correlation coefficient larger than or equal to 0.5 (*$p_ {FDR} < 0.05$*) were designated as *epispliced genes*. **(B)** Genes not possessing DHM annotations were classified as *non-epispliced* genes. The flanking regions ($\pm {200bp}$) of the alternative exons of these genes were extracted for further analysis.

rMATS(V4.2.0) [24] was used to determine differential inclusion and exclusion levels of exons of expressed genes in each cell-line pair. Skipped and mutually exclusive exons supported by junction reads were analyzed. Percent Spliced-In (PSI) values are computed as the ratio of the number of junction reads supporting the inclusion of an exon to the total number of junction reads supporting both inclusion and exclusion of that exon. This test statistic returned by rMATS was used as the DEU score. A DEU threshold of 0.2 was used to mark exons with *$|DEU| \ge 0.2$* and *$p_ {FDR} < 0.05$* as alternative exons. All other candidate exons of these differentially spliced genes were given a DEU score of 0. The usage scores of candidate exons were extrapolated to their flanks, as shown in Fig. 3, using BEDtools.

### DHM analysis

MANorm(V1.3.0) [25] was used in order to identify regions with DHMs in a pairwise manner across all available epigenomes, with default parameters. The log2 fold change of the read density (M-value) at each peak region was used as DHM score (*$p_ {FDR} < 0.05$*). Common (i.e., overlapping) peaks identified in samples of both conditions were filtered out. The intersect command from the BEDtools suite was used to annotate candidate exon flanks with the peak scores reported by MANorm. Zero imputation was performed to annotate flanks that possess no differential peaks. If more than one peak was detected in the same flank, the peak with the highest absolute DHM score was prioritized. Peaks covering exons occurring within 200 bp of transcription start sites, and also candidate exon flanks, were treated as peaks associated with transcription initiation, and their annotations were subsequently removed from the candidate exon flanks. Additionally, annotations of peaks that failed to cover at least half an exon’s length were removed. This ‘peak-leak’ filtering is illustrated in Fig. 2B.

### DEU–DHM correlation

Pearson correlation was used to measure the association between DEU and DHM scores. Taking into account the effect of sample size on correlation, genes with fewer than three flanks were filtered out beforehand. For every histone mark, genes with a strong and significant correlation $(R \ge 0.5,$  $p_ {FDR} < 0.05)$ between the annotated absolute DHM and DEU scores were obtained at the exon flanks (Fig. 3A). This group of genes was additionally filtered to ensure that DHM peaks were annotated only to flanks with non-zero DEU scores, i.e., flanks of alternative exons, and labeled as *epispliced* genes. Genes whose exon flanks were annotated with DEU scores but without DHM scores were classified as *non-epispliced* genes. The alternative exons of the epispliced and non-epispliced genes were labeled *epispliced* and *non-epispliced* exons, respectively.

### RBP binding prediction

In order to compare the behavior of RBPs at the flanks of the epispliced exons (*$DEU\ \&\ DHM$*) relative to the flanks of the non-epispliced exons (*$DEU\ \&\ \lnot DHM$*), *MaxEntScan* [26] was employed to score the 3’ and 5’ splice sites of these exons.

#### Query sequences

With the intention of elucidating the general splicing mechanism regulated by epigenetics, all flanks of epispliced exons associated with a histone mark $\it {h} \subseteq${H3K27ac, H3K27me3, H3K36me3, H3K9me3, and H3K4me3} were consolidated from all pairwise analyses of embryonic stem cells. As control, the flanks of non-epispliced exons that were reported in at least 70% of the analyses were collected. This was done to ensure there existed a set of common control exons while minimizing the effect of condition-specific splicing events. Fig. 3 illustrates this compilation.

#### Query binding motifs

RBP binding prediction was performed using RBPmap [27](V1.2). All RBPs in the internal database of RBPmap were considered. Additionally, RBPs for which eCLIP data in K562 and HepG2 cell lines were available on ENCODE3 were included. In total, this resulted in 160 RBPs. For RBPs not in the RBPmap internal database, motifs were collected from cisBP-RNA [28], mCrossBase (minimum of 50 binding sites to support the motif) [29], and other literature sources as detailed in Supplementary Table S1. For every RBP, each query exon flank was annotated with the putative binding score of the RBP in that flank. If an RBP was reported to have more than one putative binding site in a given flank, the strongest binding event was prioritized. The binding affinities were reported as Z-scores. A Z-score threshold of 2 was used to identify strong binding events, marking events with smaller affinities as non-binding events. Zero imputation was performed to handle exon flanks that had no predicted binding events for a given RBP. The sequence logos of the RBP motifs were generated using the Python library, *Logomaker* [30].

### Binary classification

For every histone mark, the feature matrix consisted of the predicted binding scores of the 160 RBPs at the considered epispliced and non-epispliced exon flanks, and the corresponding binary class labels (Supplementary Table S2). Baseline predictions were generated based on the original class stratification. The Random Forest binary classifiers and baseline models were implemented using the Python package sklearn [31]. Pearson correlation and Principal Component Analyses were performed to determine the need for feature selection. The models were run with default parameters, using the Gini impurity measure to determine optimal node splits. Class imbalance was addressed using weights inversely proportional to class frequency. A Stratified cross-validation strategy (K = 5) with 200 trees and 10 repeats per fold was chosen to ensure robustness. The models were interpreted using the SHAP values of the features. SHAP aids in understanding the model’s interpretability on a per-observation basis. Features that have high absolute SHAP values are anticipated to have a greater impact on classification. In the current study, SHAP values were used to clarify the classification of each exon flank as belonging to an epispliced or non-epispliced exon, respectively, by assessing how much the binding score of each RBP contributes to that prediction.

### Validation using eCLIP evidence

Processed eCLIP data were procured from ENCODE for the HepG2 and K562 cell lines. The epispliced exon flanks were obtained from genes with differential splicing events in the HepG2 and K562 cell lines. To ensure confidence, only DEU events reported by rMATS with a minimum of 10 reads supporting them were considered. The differential ChIP-Seq and eCLIP peaks occurring within these flanking regions were obtained using Bedtools intersect, and visualized using the Gviz [32] package. The ChIP seq alignment files were normalized relative to each other using *bamCompare* [33] to obtain coverage tracks.

### RBP expression analysis

The read summarization tool *featureCounts* [34] was incorporated to generate a count matrix of all detected genes in the five embryonic cell lines, as well as the two cancer cell lines. The varying library sizes were normalized using the TMM method from *edgeR* [35]. TMM-normalized TPM values were log_2_-transformed and visualized.

### PPI with Chromatin-regulator proteins

The proteins that were functionally associated with the five histone marks were obtained from UniProt. Additional proteins were obtained from the EpiFactors database [36]. This was done by filtering for the histone marks of interest in the *’Target’* column. The list of proteins is provided in Supplementary Table S1. PPI networks between RBPs and chromatin-associated proteins were obtained using STRING [37] using two filters: *$Interaction sources: Experiment, Interaction score \ge 0.4$*.

### Statistical analyses

For each of the five histone marks, RBPs with differential predicted binding preferences between the flanking regions of epispliced (*$DEU\ \&\ DHM$*) and non-epispliced (*$DEU\ \&\ \lnot DHM$*) exons were identified. They were termed episplicing or non-episplicing RBPs, depending on their relative binding strength. The SciPy implementation of Welch’s t-test was used to test the null hypothesis that the mean binding scores of these RBPs do not vary between the different exon classes.: epispliced exons (*$DEU\ \&\ DHM$*), non-epispliced exons (*$DEU\ \&\ \lnot DHM$*), and constitutive exons with deregulated histone signals (*$\lnot DEU\ \&\ DHM$*).

## Results

### Exon-skipping events of genes are associated with local epigenetic changes

Data from five cell lineages were analyzed, belonging to different stages of differentiation: H1 cell line, ectodermal cell, endodermal cell, mesodermal cell, and neuronal stem cell. Based on the availability of histone modification ChIP-Seq data for these biosamples in the ENCODE compendium, H3K27ac, H3K27me3, H3K36me3, H3K9me3, and H3K4me3 were chosen as the epigenetic signals of interest in this study. After preliminary filtering, a list of exons from well-supported transcripts was obtained. The tool rMATS was employed to characterize differential inclusion levels, i.e., DEU scores of these candidate exons. Then, the M-values of the ChIP-Seq peaks returned by MANorm were used as the DHM scores.

**Figure 4.**
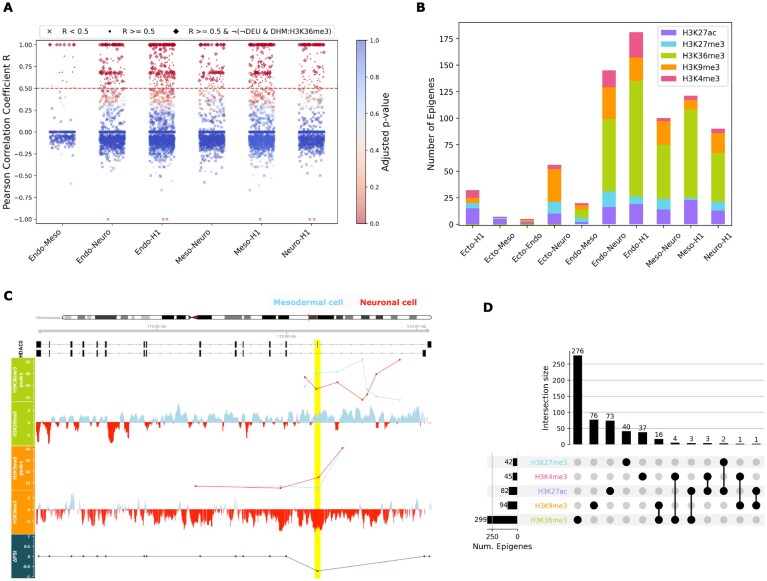
Genes whose alternative splicing events were correlated to histone modifications annotated only to alternative exons were called *epispliced genes* (*$R \ge 0.5$, $p_ {FDR} < 0.05$*); their alternative exons were called *epispliced exons*. **(A)** Manhattan plot illustrating the multistep filtering of candidate genes to obtain epispliced genes associated with H3K36me3. Genes passing ($R \ge 0.5$) and failing ($R < 0.5$) the correlation coefficient thresholding are represented using dot and cross-markers, respectively. Candidate genes additionally filtered based on alternative exon-specific DHM peak enrichment are represented using diamond markers. Supplementary Fig. S4 shows similar plots for the other histone mark-associated epispliced genes. **(B)** Number of epispliced genes found for five histone marks from all pairwise analyses across the embryonic cell lines. The number of epispliced genes associated with H3K36me3 shown here corresponds to the number of diamond markers in **A. (C)** Two transcripts of an epispliced gene, HDAC2, whose alternative exon inclusion was associated with the deregulation of H3K36me3 and H3K9me3 in the neuronal and mesodermal cell lines (highlighted in yellow). The $\Delta PSI$ score is positive for exons differentially included in the mesodermal cell. For each of the two histone marks shown, the normalized read densities of peak regions (*$p_{FDR} < 0.05$*) as reported by *MAnorm* are shown in the upper track, with the normalized read coverage obtained using *bamCompare* right below. The coverage tracks are provided to visualize the pattern of read density at the exon-intron boundaries within each biosample. Since the underlying normalization approaches of *MAnorm* and *bamCompare* differ greatly, the normalized read densities at the peak regions reported by these tools are not always concordant. **(D)** Number of epispliced genes available for each histone mark, including those reported for two different marks.

In order to identify epigenetic signals at the exon-intron boundaries, flanking regions of length $\pm$200bp were obtained for the differentially-used exons as target sequences of interest. These exon flanks were then annotated with the DEU scores of the exons they were derived from, and with the DHM scores of the histone mark peaks occurring in these regions. As conducted in a previous study [20], we grouped and correlated the two scores in a gene-wise manner and extracted those genes whose absolute DEU and DHM values were Pearson correlated such that $R \ge 0.5,$  $p_ {FDR} < 0.05;$ these resulting genes were categorized as *epispliced genes* (Fig. 4A, Supplementary Fig. S4). It was hypothesized that alternative splicing events of these genes may be regulated by the histone mark enriched in the $\pm$200bp region of the differentially spliced exons. Epispliced genes associated with each of the five histone marks were obtained in each of the 10 pairwise analyses among the five cell lines (Fig. 4B, Supplementary Table S2). While most of the identified epispliced genes were associated with a single histone modification, there were a few cases in which the DEU event(s) of the same gene coincided with two different histone signals (Fig. 4D, Supplementary Table S2). Fig. 4C shows the differential usage of an exon of gene HDAC2 between neuronal and mesodermal cells; the differential inclusion of the exon in neuronal stem cells is correlated with the differential magnitudes of the H3K36me3 and H3K39me3 peaks in the mesodermal and neuronal cell lines, respectively.

### RBP binding affinities can predict the local epigenetic state of alternative exons

**Figure 5.**
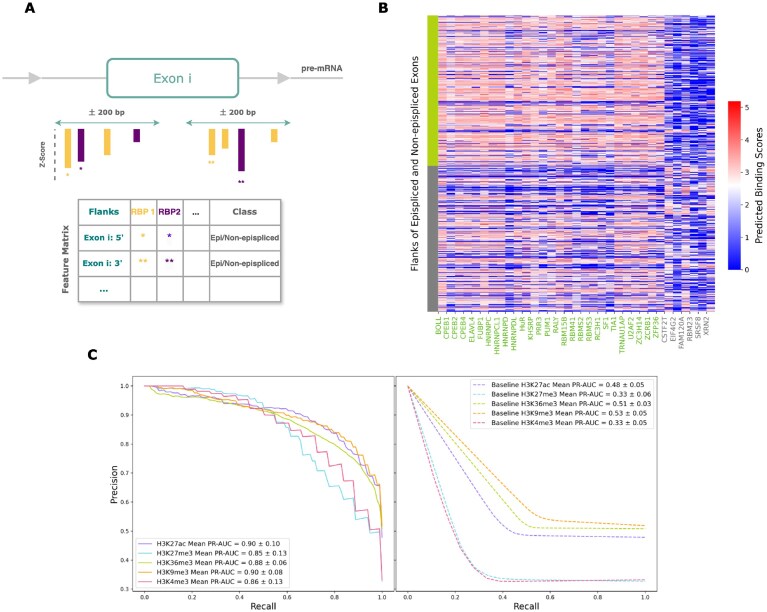
**(A)** Construction of the feature matrix. For a given exon flank, if an RBP was reported by RBPmap to have more than one putative binding site in a given flank, the strongest binding event was prioritized. **(B)** Putative binding scores of the identified episplicing and non-episplicing RBPs associated with H3K36me3. The flanking sequences of the non-epispliced exons are marked in gray, while those of the epispliced exons are highlighted in green. Likewise, the episplicing and non-episplicing RBPs associated with H3K36me3 are annotated in green and grey, respectively. **(C)** Performance of the random forest binary classifiers in comparison with baseline models.

Next, the binding preference of RBP at the flanking regions of the alternative exons of the epispliced genes, the epispliced exons, was investigated. For comparison, *non-epispliced genes* were defined as those whose alternative splicing events were not associated with the chromatin state; the flanking regions of their alternative exons were treated as controls. For each histone mark, the epispliced and non-epispliced exon flanks were combined across the ten pairwise analyses into a single dataset, without overlaps between the classes. This combinatorial approach enables the study of episplicing in a developmental context, while also handling the disadvantage of data insufficiency in individual pairwise comparisons. As shown in Supplementary Table S1, histone ChIP-Seq data were available for H3K27ac, H3K27me3, H3K4me3, and H3K9me3 in all cell lines, resulting in a common set of non-epispliced exon flanks. Meanwhile, ChIP-Seq data were not available for H3K36me3 in the ectodermal cell line, resulting in episplicing analysis only being carried out for six pairwise analyses, as opposed to 10, explaining its elevated number of non-epispliced exon flanks relative to the other marks (Supplementary Fig. S1A). Then, the tool RBPmap was used to predict putative binding positions of 160 RBPs on the epispliced and non-epispliced exon flanks (Fig. 5A). When visualizing the correlation among the RBPs based on their putative binding strengths, we observed that in addition to the proteins from the same family (CPEB, KHDRBS, RBMS, PABPC), some proteins with similar binding motifs showed strong association with each other (Supplementary Fig. S5). As there were only a few such correlated RBPs, feature selection was not performed. This choice was further supported by the inability of the top principal components to capture most of the variability in the data (Supplementary Fig. S6).

Using the RBPmap-derived binding scores of the 160 RBPs on these exon flanks, random forest classifiers were trained to classify flanks as derived from either epispliced or non-epispliced exons (Supplementary Fig. S1A, Supplementary Table S2). Based on the PR-AUC metric, the optimistic performance of the models indicated the ability to distinguish the flanks of epispliced exons from non-epispliced exons based on the binding affinities of RBPs (Fig. 5C). The datasets of the H3K27me3 and H3K4me3 models were skewed toward the negative class (Supplementary Fig. S1A); the effect of this class imbalance is reflected by their specificity and recall scores (Supplementary Fig. S1B). The recall-precision trade-off of these models shows that these models were highly cautious. As this study prioritizes the accurate prediction of epispliced exon flanks, an emphasis was placed on minimizing false positives over false negatives. We proceeded to interpret these models despite their low recall, as they demonstrate reasonable precision (Supplementary Figs S1B and S7).

### RBPs preferentially bind to skipped exons marked by a histone mark

**Figure 6.**
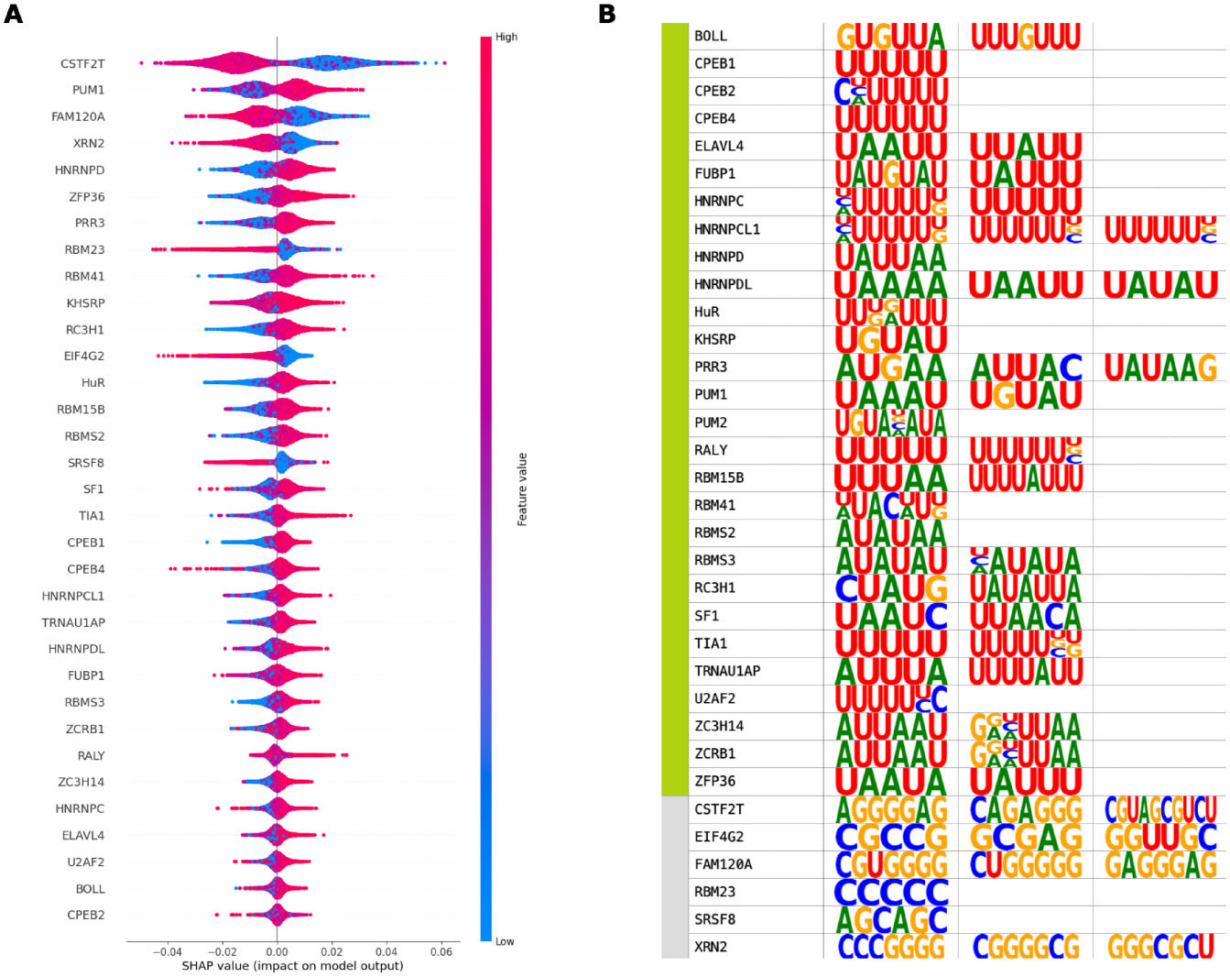
**(A)** SHAP values of the RBPs are positively and negatively associated with H3K36me3. Mean SHAP values of the RBPs were used to ascertain key model predictors, such that the RBPs are inferred to bind preferentially to either epispliced or non-epispliced exon flanks. RBPs whose predicted binding was associated with a strong, positive SHAP value in most flanking regions of epispliced exons were classified as *episplicing RBPs*, while those with marked weak binding in epispliced exon flanks were labeled as *non-episplicing RBPs*. The list of episplicing and non-episplicing RBPs was expanded to include the RBPs that were strongly correlated to them (*R >= 0.7, p_FDR_ < 0.05*). SHAP plots of the other histone-mark features are shown in Supplementary Fig. S9. **(B)** Binding motifs of episplicing RBPs associated with H3K36me3 (in green) are predominantly AU-rich, while the non-episplicing RBPs (in gray) displayed GC-richness. Supplementary Fig. S14 shows the binding motifs of the other histone mark-associated proteins.

Key features that contributed to model performance were identified, which proved challenging, considering that RBP binding events could not be treated as independent occurrences. SHAP values of the RBPs were used as the basis to interpret the model, to identify which RBPs play a crucial role in successfully distinguishing the exon flanks between the two categories. The RBPs with preferential predicted binding to flanks of epispliced exons relative to the non-epispliced exons were identified, see Fig. 6A. The model prediction for each exon flank is influenced by the magnitude of correlation between the features. RBPs with similar constituent binding domains and motifs exhibit comparable binding affinities [38]. This results in highly correlated RBPs, which may be treated as redundant features and attribute low importance by the model [39]. Therefore, to avoid discarding relevant RBPs, Pearson correlation was computed to find those whose binding scores were strongly correlated to those of the RBPs selected using the SHAP scores $(R \ge 0.7,$  $p_ {FDR} < 0.05).$ These resulting RBPs were classified as *episplicing RBPs* (Supplementary Table S3). Adding correlated RBPs increased the number of episplicing RBPs by 1, 17, 9, and 14 for H3K27me3, H3K36me3, H3K9me3, and H3K4me3, respectively (Supplementary Fig. S10).

Additionally, the same method above was used to identify RBPs whose binding events were predictive of non-epispliced exons. By definition, these *non-episplicing RBPs* were expected to preferentially bind to alternative exons not marked by any of the five histone marks. The overlap between the non-episplicing RBPs obtained from the five histone-mark models was minimal (Supplementary Table S4). This implied that their splicing preferences were histone-mark specific, such that non-episplicing RBPs identified from a specific histone mark model were predicted to exhibit weak binding to the flanking regions of the alternative exons, which are marked by that chromatin modification. This was reiterated by the overlaps between the episplicing RBPs and non-episplicing RBPs of different models. For instance, there were overlaps between episplicing RBPs from the H3K36me3, H3K9me3, and H3K4me3 models with the non-episplicing RBPs from the H3K27me3 model, and vice versa (Supplementary Tables S3 and S4, Supplementary Fig. S14). This suggests that for each histone mark, the binding events of the episplicing and non-episplicing RBPs were to be interpreted within the bounds of the classifier pertaining to that histone mark.

Alternatively-included exons have relatively weaker splice sites than constitutive exons [40, 41]. Accordingly, a difference in predicted splice site strengths between the epispliced and non-epispliced alternative exons was not observed (Supplementary Fig. S8). However, core spliceosomal RBPs were predicted to exhibit a differential preference for either the epispliced or non-epispliced exons. For instance, U2AF2 was predicted as an episplicing RBP associated with H3K36me3 and H3K4me3, and as a non-episplicing RBP of H3K27me3-marked exons (Supplementary Tables S3, S4). The recognition and binding of U2AF2 at 3’ splice sites is frequently observed [42]. This RBP’s tendency for non-specific binding suggests that it may be regarded as an artifact. It could also be argued that it is categorized as an episplicing RBP because of its RNA-binding motif resemblance to other predicted key model features (Fig. 8). However, previous studies have connected spliceosomal proteins to episplicing; it has been shown that the spliceosome interacts with the H3K4me3 reader CHD1 via member proteins of the U2-snRNP complex [43]. Additionally, a recent study has shown the increased inclusion of exons affected by U2AF2 interacting with H3K36me3-annotated chromatin [44].

**Figure 7.**
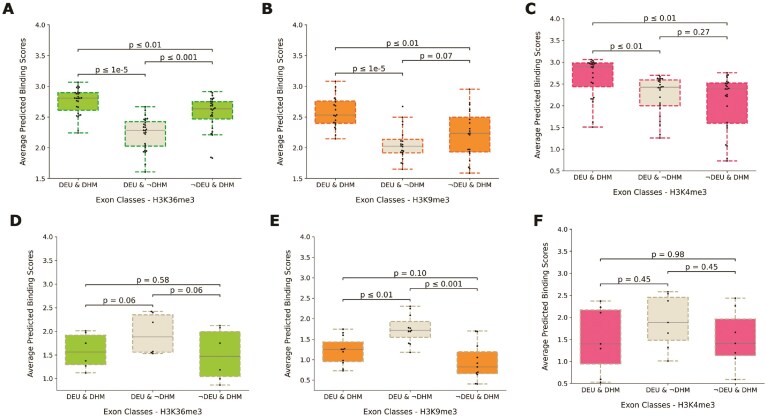
For each of the five histone marks, RBPs with differential predicted binding preferences between the flanking regions of epispliced (*$DEU\ \&\ DHM$*) and non-epispliced (*$DEU\ \&\ \lnot DHM$*) exons were identified. They were termed episplicing or non-episplicing RBPs, depending on their relative binding strength. The distributions of their mean binding affinities at exon-flanking regions were compared across three classes of exons: epispliced (*$DEU\ \&\ DHM$*), non-epispliced (*$DEU\ \&\ \lnot DHM$*), and constitutive exons with deregulated histone signals (*$\lnot DEU\ \&\ DHM$*). **(A**–**C)** show the binding score distributions of episplicing RBPs predicted to associate with H3K36me3, H3K9me3, and H3K4me3, respectively. **(D–F)** show the binding score distributions of non-episplicing RBPs predicted to show weak binding to alternative exons marked by H3K36me3, H3K9me3, and H3K4me3, respectively. Supplementary Fig. S13 shows the distribution plots for H3K27ac and H3K27me3.

**Figure 8.**
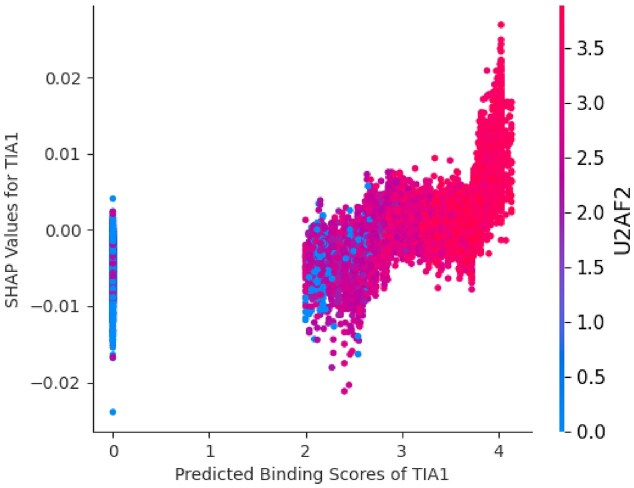
SHAP scores of TIA1 reported in the H3K36me3 model are plotted against its predicted binding scores at the epispliced and non-epispliced exon flanks. The plot also displays the similarity of the binding score distribution between TIA1 and U2AF2.

### Sequence specificity of histone mark enrichment around exons

Then, the binding motifs of the episplicing and non-episplicing RBPs associated with each histone mark were compared. Fig. 6B shows that episplicing RBPs associated with H3K36me3 are enriched in AU-rich motifs, while the binding motifs of non-episplicing RBPs were GC-rich. This proved similar for the RBPs associated with H3K9me3 and H3K4me3 (Supplementary Fig. 14C and D), as expected, considering the large overlap in the episplicing and non-episplicing RBPs between the three marks (Supplementary Tables S3 and S4). This also supports the possible combinatorial regulation of splicing by these histone marks, as hinted in Fig. 4D. To some extent, the RNA motif enrichment was inverted in H3K27me3 (Supplementary Fig. S14B) with the episplicing and non-episplicing RBPs showing an enrichment of GC and AU motifs, respectively. This cross-overlap between episplicing and non-episplicing RBPs of H3K36me3, H3K9me3 and H3K4me3, and H3K27me3 was previously discussed (Supplementary Tables S3 and S4). It was difficult to verify a principal motif choice among the episplicing RBPs of H3K27ac (Supplementary Fig. S14A). This could be explained by the poor correlation between the binding events of these RBPs (Supplementary Fig. S10C).

Based on these findings, a question arises of whether RBP binding affinities alone suffice to classify exon flanks. Upon visualizing the feature matrices of the five models as heatmaps, it became apparent that the RBPs do not exhibit an exclusive preference for either sequence class. However, episplicing and non-episplicing RBPs associated with H3K36me3 and H3K9me3 are enriched in strong and weak binding strengths at their respective epispliced exon flank sequences (Fig. 5B, Supplementary Fig. 11C). No similar pattern of differential binding affinity was detected for the episplicing and non-episplicing RBPs associated with H3K27ac, H3K27me3, and H3K4me3 (Supplementary Fig. S11A, S11B, S11D). Another contributing factor was the imbalance in the datasets of H3K27me3 and H3K4me3 (Supplementary Fig. S1A), which complicated determining the differential preference of the RBPs between the two classes, based solely on visualization. Welch’s t-test was employed to compare the binding scores of the episplicing and non-episplicing RBPs obtained for each histone mark model between the flanking sequences of the epispliced and non-epispliced exons (Supplementary Fig. S12). A significant difference ($p_{FDR} \le 0.05$) in binding strength distributions of episplicing RBPs associated with H3K36me3, H3K9me3, and H3K4me3 between the flanking sequences of the two exon types was found. Additionally, the binding score distribution of the non-episplicing RBPs associated with H3K27ac, H3K27me3, and H3K9me3 also varied significantly ($p_{FDR} \le 0.05$) between the two exon classes.

Considering that the performance of each classifier is attributed to the predictive power of its important features, i.e., the episplicing and non-episplicing RBPs, it was expected that the performance of the classifier would deteriorate without the information provided by these features. Surprisingly, that was not the case. Upon observing the most important features of these new models (Supplementary Fig. S15), a similarity in their motifs was revealed, namely regarding those of the episplicing and non-episplicing RBPs reported by the original model (Fig. 6, Supplementary Fig. S14). This restates the presence of certain binding motifs in the vicinity of exons enriched with differential epigenetic modifications.

Consequently, it was expected that these predicted episplicing RBPs would bind neighborhoods of exons marked by deregulated histone signals regardless of the inclusion status of the exons. In order to further study this, exons with local differential histone peaks were collected for constitutively spliced genes (*$\lnot DEU\ \&\ DHM$*). The binding score distributions of the episplicing and non-episplicing RBPs were compared using Welch’s t-test across the flanking regions of these exons, along with those of epispliced (*$DEU\ \&\ DHM$*) and non-epispliced (*$DEU\ \&\ \lnot DHM$*) exons (Fig. 7, Supplementary Fig. S13, Supplementary Table S2). Interestingly, the binding scores of the episplicing RBPs associated with H3K36me3, H3K9me3, and H3K4me3 were stronger at the regions surrounding the alternative (epispliced) exons relative to the constitutive exons ($p_{FDR} \le 0.01$). However, they show stronger putative binding at the flanks of the constitutive exons than those of non-epispliced exons in the case of H3K36me3 ($p_{FDR} \le 0.001$), suggesting a bias of H3K36me3 deposition at AU-rich sequence motifs. AU-rich regions are commonly found in the 3’UTR regions of mRNAs [45]. The enrichment of H3K36me3 at 3’UTR elements [46, 47] further supports this supposed preference. Furthermore, RBPs that bind these regions play roles in 3’UTR-linked RNA biogenesis and splicing [48].

The non-episplicing RBPs associated with H3K9me3, H3K27ac, and H3K27me3 exhibit significantly stronger putative binding at the exon-intron regions of non-epispliced exons relative to the alternative and constitutive exons with DHM annotations ($p_{FDR} \le 0.001$). While the binding scores of the episplicing RBPs associated with H3K27me3 do not differ significantly between alternative (epispliced) and constitutive exons (Supplementary Fig. S13B), the binding pattern of non-episplicing RBPs is significantly stronger at the flanks of epispliced exons relative to constitutive exons with differential histone signal annotations ($p_{FDR} \le 0.001$) (Supplementary Fig. S13D).

### Episplicing in HepG2-K562 cell lines

Tissue-specific epigenetic states tailor the transcriptional profiles in corresponding tissues [49, 50]. We wanted to determine if these findings were specific to the embryonic cell lines or could apply to other tissues as well. To that extent, we analyzed whether eCLIP peaks were detected for any of the predicted episplicing RBPs within $\pm$ 200 bp of alternative exons tagged by the histone marks of interest in each of the two cancer cell lines, K562 and HepG2. There was no ChIP-Seq data available for H3K27me3 in the HepG2 and K562 cell lines. Hence, the eCLIP evidence of the episplicing RBPs predicted to associate with H3K27me3 could not be reviewed. Notably, only 11 of the 47 proteins for which eCLIP data were available were reported as episplicing RBPs. Specifically, eCLIP data could be analyzed for 4, 5, 1, and 7 episplicing RBPs, whose binding was predicted to associate with H3K27ac, H3K36me3, H3K9me3, and H3K4me3, respectively (Supplementary Tables S1, S3). The expression levels of all predicted episplicing RBPs are shown in Supplementary Fig. S18.

As expected, the epispliced genes associated with H3K36me3 were overrepresented relative to the other marks (Supplementary Fig. S3A). TIA1 and U2AF2 were predicted to associate with H3K36me3 (Fig. 8); eCLIP peaks of these two RBPs were found in the vicinity of the epispliced exons acquired from the HepG2-K562 cell-line pair. A total of 5885 high-confidence peaks for TIA1 in K562 and 10,732 peaks for U2AF2 in HepG2 were identified from ENCODE3. Assuming a uniform genomic distribution of these eCLIP peaks, the probability of detecting at least one peak within a 200 bp exon flank window is highly significant, with *P*-values of $3.923\times 10^{-4}$ for TIA1 and $7.154\times 10^{-4}$ for U2AF2. Experimental evidence of direct interaction between these RBPs and proteins associated with H3K36me3 was not found in the current literature. The coordination of the splicing, epigenetic, and RBP-binding signals for two genes can be observed in Table 1; inspecting the table, there are varying patterns between exon usage, histone modifications, and RBP binding between K562 and HepG2, which can be categorized into three cases.

**Table 1.** For certain episplicing RBPs predicted in embryonic cell lines, eCLIP peaks were detected in a 200 bp window around epispliced exons identified in K562 and HepG2. For these two cell lines, epispliced genes were identified based on their RNA-Seq and ChIP-Seq data, as before. This returned 6, 29, 1, and 3 genes for H3K27ac, H3K36me3, H3K9me3, and H3K4me3, respectively (Supplementary Fig. S3A, Supplementary Table S2)

Gene	Exon coordinates	DEU	DHM	eCLIP	Case
CD46	chr1:207790253-207790345	K562	H3K36me3 K562	TIA1 K562	Case 1
MAP3K8	chr10:30437176-30437406	HepG2	H3K36me3 K562	TIA1 K562	Case 2
TMTC4	chr13:100656381-100656468	K562	H3K36me3 HepG2	U2AF2 HepG2	Case 2
ARF4	chr3:57577316-57577387	HepG2	H3K36me3 HepG2	U2AF2 K562	Case 3
CD46	chr1:207790253-207790345	K562	H3K36me3 K562	U2AF2 HepG2	Case 3
PUS7	chr7:105468337-105468463	K562	H3K36me3 K562	U2AF2 HepG2	Case 3
RPS6KB1	chr17:59912684-59912804	K562	H3K36me3 K562	U2AF2 HepG2	Case 3

#### Case 1: Exon inclusion is positively correlated with the histone mark and eCLIP peaks

**Figure 9.**
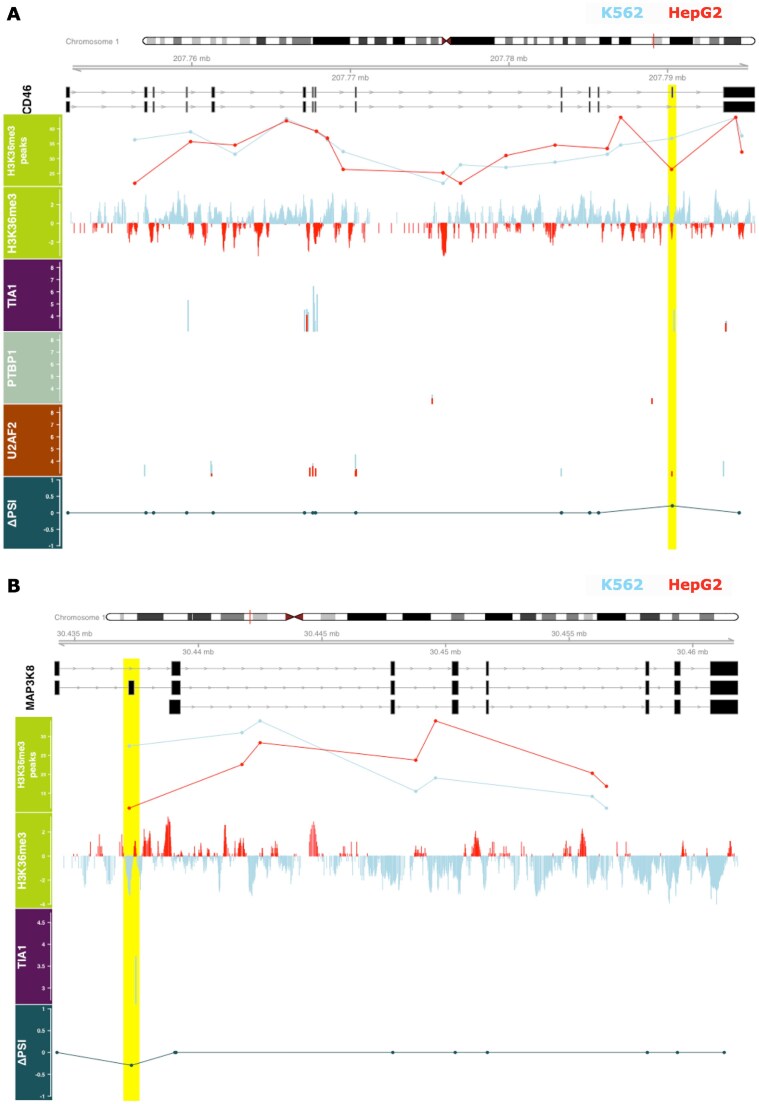
**(A)** An alternative exon of CD46 is marked by H3K36me3 and bound by TIA1 and U2AF2. **(B)** An exon of MAP3K8 is differentially included and marked by H3K36me3 in HepG2, but bound by TIA1 in K562. The differential exon extended by 200 bp on both sides is highlighted in yellow. The $\Delta PSI$ score is positive for exons differentially included in the K562 cell line and negative for exons differentially included in the HepG2 cell line. Few transcripts are shown for ease of visualization. The normalized read densities of H3K36me3 peaks (*$p_{FDR} < 0.05$*) as reported by *MAnorm* are shown in the first track, followed by the normalized read coverage obtained using *bamCompare* right below. The coverage tracks are provided to visualize the pattern of read density at the exon-intron boundaries within each biosample. Since the underlying normalization approaches of *MAnorm* and *bamCompare* differ greatly, the normalized read densities at the peak regions reported by these tools are not always concordant. Sashimi plots displaying RNA-Seq densities at exon and junction regions are shown in Supplementary Figs S16 and S17.

In this case, the histone mark is assumed to regulate the splicing of these exons by interacting with RBPs binding to a $\pm$200 bp window around them. For instance, CD46 is a member of the immune complement system, with roles in both innate and adaptive immune responses [51]. The differential inclusion of exon 13 in its mRNA results in two non-identical protein products with varying C-terminal domains, leading to dissimilar molecular signaling characteristics [52, 53]. Increased inclusion of this exon was detected in the K562 cell line (Fig. 9A) in concordance with the enrichment of an H3K36me3 peak marking this exon, along with an eCLIP peak for TIA1, a predicted episplicing RBP, at the 3’ exon flank. TIA1 was shown to influence the recruitment of the U1 snRNP to the 5’ splice sites by binding the downstream neighboring regions of the splice sites [54–56]. Tang et al. conducted knockdown and overexpression assays to characterize the role of specific RBPs in the inclusion of this exon in HeLa, HEK293T, and Jurkat cell lines [57]. Interestingly, it was found that TIA1 and TIAL1 promote exon inclusion, while SRSF1 and PTBP1 suppress it. As eCLIP peak evidence was available for the two repressor proteins, their binding around this exon could be examined. While the study by Tang et al. postulated that PTBP1 promotes exon 13 exclusion by binding its exonic silencing element, an eCLIP peak of PTBP1 was detected upstream of exon 13 in HepG2 cells, where the exon usage is downregulated. No similar peaks of SRSF1 were found in either cell line. In mesenchymal cells, the binding of PTBP1 has been linked to H3K36me3-associated exclusion of exon 3b of the gene FRGR2 [10]. Additionally, SRSF1 interacts with a H3K36me3 reader, Psip1 [11]. Wang et al. observed that TIA1 and TIAL1 bind the same regions, using iCLIP data in HeLa cells [58].

Altogether, these findings imply a possible epigenetic regulatory mechanism underlying the inclusion of exon 13 of CD46.

#### Case 2: Exon inclusion is negatively correlated with the histone mark and eCLIP peaks

The histone mark is now assumed to inhibit splicing of these exons by interacting with RBPs binding to a $\pm$200bp window around them. Exon 2 of the gene MAP3K8 is differentially included in the HepG2 cell line. However, a H3K36me3 ChIP-Seq peak marks the exon in the K562 cell line, along with a downstream TIA1 peak (Fig. 9B). MAP3K8 is an oncogene and kinase that participates in the inflammatory response of various conditions [59–61]. The protein diversity of MAP3K8 is attributed to two alternate translation initiation sites [62]. The downstream effect of the differential usage of exon 2 is not extensively reported. Contrary to the discussion above, in this context, the binding of TIA1 seems to be associated with decreased exon inclusion. The dual role of TIA1 in affecting exon usage has been presented by previous studies, as well [58, 63].

#### Case 3: eCLIP peak is negatively correlated with the histone mark and exon usage

Here, binding of U2AF2, another splicing regulator predicted as an episplicing RBP (Supplementary Table S3), was detected at the 5’ region of the 13th exon of CD46, overlapping the intron-exon boundary in the HepG2 cell line (Fig. 9A). It is a subunit of a heterodimer that is part of the snRNP U2 Auxiliary Factor complex, which recognizes conserved splicing signals at the 3’ splice site of an intron, facilitating assembly of the spliceosomal machinery at this region. Knockdown studies of the RBP have shown both increased and decreased inclusion of skipped exons [42, 64], extending its role to include splicing repression.

Splicing events in which a histone mark was negatively correlated with exon usage and eCLIP peaks were also identified. Here, the histone mark is assumed to inhibit splicing of these exons by regulating the transcriptional kinetics or interacting with RBPs that do not possess eCLIP data on ENCODE3. All usage events of epispliced exons from the K562-HepG2 comparison under these categories are listed in Supplementary Table S6.

The previous section suggested a predisposition of H3K36me3 enrichment at AU-rich regions (Fig. 7A). In the K562-HepG2 comparison, out of 21 constitutive exons with deregulated H3K36me3 annotations, 3 exons were annotated with differential U2AF2 eCLIP peaks within a $\pm 200bp$ boundary (Supplementary Table S7). The pattern of U2AF2 binding was positively correlated with DHM occurrence in two of these exons. Additionally, eCLIP peaks were also reported for EFTUD2 in the vicinity of three exons; they were positively correlated with H3K36me3 differential peak enrichment. While EFTUD2 is not identified as an episplicing RBP in this study, it has a binding motif characteristic of episplicing RBPs of H3K36me3 (Supplementary Fig. S15F). The H3K36me3 recognition protein ZMYND11 and the RBP EFTUD2 form an adapter complex regulating intron retention [12].

## Discussion

In this study, two classes of alternative exons were identified: those differentially co-occurring with at least one mark among H3K27ac, H3K27me3, H3K36me3, H3K9me3, and H3K4me3, and those marked by neither of these marks. The putative binding affinities of 160 RBPs at the exon-intron boundaries of these exons were obtained. Importantly, to date, only a subset of these RBPs possess functional annotations related to splicing. The remaining RBPs are yet unconnected to splicing, implicated in mRNA metabolism, or remain poorly characterized. Biological data is often characterized by high dimensionality. Based on the effectiveness of Random Forests in handling $p >> n$ datasets [65], binary classifiers were trained for each of the five histone modifications considered in this study to predict the class of an alternatively-spliced exon, based on putative binding scores of our candidate RBPs at its 5’ and 3’ flanking regions. Stringent filtering criteria were applied to identify high-confidence episplicing events, which yielded a focused dataset for each model. To ensure robustness of the models, we did not further distinguish whether certain RBPs were predicted to bind upstream or downstream of a specific exon; yet it is known that some RBPs exert different effects depending on their binding position relative to the exon [66]. It was examined whether the epigenetic signals occurring in the transcription termination neighborhood could have influenced the positive performance of the classifiers. However, the 3’ terminal exons were underrepresented in the compiled groups of epispliced exons (Supplementary Fig. S2). Using their SHAP measures, relatively important features were obtained and classified as either episplicing or non-episplicing RBPs. Upon inspecting the binding motifs of these RBPs, the differential binding preference of certain protein-binding motifs could be discerned at the exonic neighborhoods ($\pm 200bp$) marked by the epigenetic signals of interest. Not only were the average putative binding strengths of predicted episplicing RBPs associated with H3K36me3, H3K9me3, and H3K4me3 stronger at the flanking regions of epispliced exons relative to non-epsipliced exons, but also when compared to constitutive exons with differential signal enrichment of the same histone mark. Finally, eCLIP data from ENCODE in the HepG2 and K562 cell lines were used to support TIA1 and U2AF2 as potential episplicing RBPs, as predicted by our model in the embryonic cell lines.

Epigenetic modifications have been indicated in regulating local splicing events through their influence on the kinetics of transcription, by affecting co-transcriptional splicing events, or by means of protein-protein interactions (PPI) between splicing factors and chromatin-reader proteins [67]. Experimental evidence supporting direct PPIs between chromatin-associated proteins and the predicted episplicing RBPs was found using STRING (Supplementary Table S5), suggesting potential adaptor mechanisms involved in embryonic epigenetically-regulated alternative splicing events. The confidence of these interactions, as reported by STRING, ranged from moderate (0.5) to strong ($\ge$ 0.7). Evidence of interactions between these reader proteins and non-episplicing RBPs exists, as well (Supplementary Table S5). BRD4 is a bromodomain-containing protein that functions in maintaining chromatin structure and recognizing histone acetylation tags [68]. It co-purifies with XRCC6, a poorly characterized RBP implicated in RNA editing [69, 70]. This RBP also interacts with CREBP, a histone acetyltransferase [71], and also with the core histone protein H31. RALY or HNRPCL2 regulates alternative splicing [72, 73]. This RBP also forms a PPI with BRD1, which acts as a weak H3K36me3 reader [74]. A recent investigation suggested that TRNAU1AP may function as a splicing factor in this context [75]. Here, TRNAU1AP was identified as an episplicing factor associated with H3K36me3, H3K9me3, and H3K4me3 (Supplementary Table S3). Studies in yeast show that this RBP interacts with SETD2, a H3K36 methyltransferase; SETD2 was shown to deposit H3K36me3 in a co-transcriptional manner [76, 77]. Furthermore, Almeida et al. inhibited the splicing of select genes and subsequently noticed a reduced recruitment of SETD2 [78], implying that there is a bidirectional exchange of information between post-transcriptional processing events and the epigenetic environment. The interaction between yeast homologs of TRNAU1AP and PHF5A has also been studied. Interestingly, PHF5A has roles in splicing, transcription, and the post-translational modification of histones. It is a member of the U2 snRNP spliceosomal complex. PHF5A also regulates RNA Pol II elongation and deposition of H3K36me3 marks by interacting with the PAF1C complex [79]. RC3H1, an RBP with an unknown role in splicing, interacts with TRIM28, a recruiter of H3K9me3 writer SET proteins [80], which also interacts with HP1 [81], a reader protein of H3K9me3. KHDRBS1 binds RNA with roles in alternative splicing [82], additionally interfacing with TRIM28. We postulate that H3K36me3 may act as a switch controlling the binding of RBPs, which in turn may regulate splicing outcomes (Fig. 7A, Supplementary Table S7).

Luco et al. reported that skipped exons with weaker PTB-binding sites were prone to the influence of an adaptor complex comprised of the splicing factor PTBP1 and the chromatin reader protein MRG15, bridging exon usage with local H3K36me3 modifications [10]. This may account for PTBP1 not being reported as an episplicing RBP within this study, considering that the binding strengths of RBPs were used as a positive indicator, bridging the splicing events of alternative exons with their adjacent epigenetic state. Comparing the binding scores, PTBP1 was moderately correlated with some of the identified H3K36me3-associated episplicing RBPs ($\overline{R} \approx 0.5, p_ {FDR} < 0.05$). PTBP1 is predicted, however, as an episplicing RBP associated with H3K4me3 (Supplementary Figs S9D and S14D). The Psip1-SRSF1 complex was shown to regulate the inclusion of exons in the vicinity of H3K36me3 deregulation [11]. However, the binding scores of SRSF1 were poorly correlated with the identified reported episplicing RBPs ($\overline{R} \approx 0.3, p_ {FDR} < 0.05$). The findings of this study are not intended to restrict the components of the (epi)splicing control module solely to the important RBPs identified by the classifiers. Instead, we recommend using the shared characteristics of the binding motifs of these RBPs as a framework to uncover an additional layer of splicing-associated regulatory sequence signals. For instance, Yearim et al. demonstrated that HP1, a chromodomain protein that recognizes H3K9me3, interacts with members of the U2 snRNP [83]. While U2AF2 was identified here as an episplicing RBP associated with H3K36me3 and H3K4me3, the motif similarity among the episplicing RBPs of H3K9me3, with these two marks, supports the possibility of U2AF2 functioning as an episplicing RBP of H3K9me3, as well.

The potential epigenetic regulation of microexon inclusion has also been reported within the scope of this study. These exons are ~30 nucleotides in length [84] and have been strongly associated with neuronal splicing events during development [85, 86], as well as in some neurological pathologies [87]. While the mean lengths of the epispliced exons ranged between 125 and 140 base pairs (Supplementary Fig. S3B), nine such epispliced microexons were detected (Supplementary Table S8). Four of these exons were differentially used in the neuronal cell line. Carlo et al. indicated the role of SF1 in microexon definition, whereby it increases recognition of upstream exons [88]. Remarkably, SF1 was identified here as an episplicing RBP associated with H3K36me3. Another study has notably reported regulatory roles of PTBP1 and RBFOX in microexon usage [89]. As mentioned previously, PTBP1 is an RBP known to bridge exon-specific H3K36me3 enrichment with differential inclusion of the exon [10]. RBFOX1 was predicted here as an episplicing RBP associated with H3K27ac (Supplementary Figs S11A, S14A).

The approaches used to observe the epigenetic regulation of splicing outcomes in this study have some caveats. A window size of 200 bp was used to identify deregulated histone signals at the exon boundaries. The splicing of non-epispliced exons is hypothesized to be independent of the epigenetic state since they lack deregulated histone peaks in their 200 bp-long flanks. However, there might be differential histone signals outside this fixed window [19] that may regulate the splicing of these exons. A smaller window offers a more confident view of episplicing events at the cost of missing long-range interactions. In other cases, filtering approaches may lead to the exclusion of valid episplicing events. For instance, histone modification peaks localizing within a 200 bp window to transcription start sites were treated as those functionally associated solely with transcription. However, these differential modification events could play dual roles in transcriptional and splicing regulation. It is also worth mentioning that RBPmap uses a sequence similarity-based approach to predict RNA-RBP crosstalk, without considering the influence of RNA structural conformation on the strength of these predicted interactions. Alternative tools that utilize predicted [90–92] or experimental [93] RNA structural information along with the sequence features may offer more precise RBP-binding predictions.

By focusing on coordinated changes in exon usage and histone modifications in embryonic cell lines, epigenetic regulation of splicing events in differentiation could be examined in greater detail. Specifically, histone mark-specific patterns were observed among the regulatory elements comprising exon-intron junctions. Exons marked individually or in combination by H3K36me3, H3K9me3, and H3K4me3 exhibited similar RBP-binding motifs. Future work would require similar investigations in other cellular contexts to verify if these findings are specific to embryonic differentiation or are part of a general splicing mechanism.

## Supplementary Material

lqaf161_Supplemental_Files

## Data Availability

RNA-Seq, ChIP-Seq, and eCLIP data used in this study were obtained from the ENCODE resource at https://www.encodeproject.org/. The list of biosamples and their accession IDs used in this study can be found in Supplementary Table S1. The analysis code is accessible via https://doi.org/10.5281/zenodo.17242005.

## References

[1] Bannister AJ, Kouzarides T. Regulation of chromatin by histone modifications. Cell Res. 2011;21:381–95. 10.1038/cr.2011.22. 21321607 PMC3193420

[2] Spies N, Nielsen CB, Padgett RA et al. Biased chromatin signatures around polyadenylation sites and exons. Mol Cell. 2009;36:245–54. 10.1016/j.molcel.2009.10.008. 19854133 PMC2786773

[3] Schwartz S, Meshorer E, Ast G. Chromatin organization marks exon-intron structure. Nat Struct Mol Biol. 2009;16:990–5. 10.1038/nsmb.1659. 19684600

[4] Andersson R, Enroth S, Rada-Iglesias A et al. Nucleosomes are well positioned in exons and carry characteristic histone modifications. Genome Res. 2009;19:1732–41. 10.1101/gr.092353.109. 19687145 PMC2765275

[5] Tilgner H, Nikolaou C, Althammer S et al. Nucleosome positioning as a determinant of exon recognition. Nat Struct Mol Biol. 2009;16:996–1001. 10.1038/nsmb.1658. 19684599

[6] Saint-André V, Batsché E, Rachez C et al. Histone H3 lysine 9 trimethylation and HP1$\gamma$ favor inclusion of alternative exons. Nat Struct Mol Biol. 2011;18:337–44. 10.1038/nsmb.1995. 21358630

[7] Alló M, Buggiano V, Fededa JP et al. Control of alternative splicing through siRNA-mediated transcriptional gene silencing. Nat Struct Mol Biol. 2009;16:717–724. 10.1038/nsmb.1620. 19543290

[8] Schor IE, Rascovan N, Pelisch F et al. Neuronal cell depolarization induces intragenic chromatin modifications affecting NCAM alternative splicing. Proc Natl Acad Sci. 2009;106:4325–30. 10.1073/pnas.0810666106. 19251664 PMC2657401

[9] Zhou HL, Hinman MN, Barron VA et al. Hu proteins regulate alternative splicing by inducing localized histone hyperacetylation in an RNA-dependent manner. Proc Natl Acad Sci. 2011;108:E627–35. 10.1073/pnas.1103344108. 21808035 PMC3169152

[10] Luco RF, Pan Q, Tominaga K et al. Regulation of alternative splicing by histone modifications. Science. 2010;327:996–1000. 10.1126/science.1184208. 20133523 PMC2913848

[11] Pradeepa MM, Sutherland HG, Ule J et al. Psip1/Ledgf p52 binds methylated histone H3K36 and splicing factors and contributes to the regulation of alternative splicing. PLoS Genet. 2012;8:e1002717. 10.1371/journal.pgen.1002717. 22615581 PMC3355077

[12] Guo R, Zheng L, Park JW et al. BS69/ZMYND11 reads and connects histone H3. 3 lysine 36 trimethylation-decorated chromatin to regulated pre-mRNA processing. Mol Cell. 2014;56:298–310. 10.1016/j.molcel.2014.08.022. 25263594 PMC4363072

[13] Gonzalez I, Munita R, Agirre E et al. A lncRNA regulates alternative splicing via establishment of a splicing-specific chromatin signature. Nat Struct Mol Biol. 2015;22:370–6. 10.1038/nsmb.3005. 25849144 PMC6322542

[14] Segelle A, Nunez-Alvarez Y, Oldfield AJ et al. Histone marks regulate the epithelial-to-mesenchymal transition via alternative splicing. Cell Rep. 2022;38:110357. 10.1016/j.celrep.2022.110357. 35172149

[15] Hu Q, Kim EJ, Feng J et al. Histone posttranslational modifications predict specific alternative exon subtypes in mammalian brain. PLoS Comput Biol. 2017;13:e1005602. 10.1371/journal.pcbi.1005602. 28609483 PMC5487056

[16] Xu Y, Wang Y, Luo J et al. Deep learning of the splicing (epi) genetic code reveals a novel candidate mechanism linking histone modifications to ESC fate decision. Nucleic Acids Res. 2017;45:12100–12. 10.1093/nar/gkx870. 29036709 PMC5716079

[17] Lee D, Zhang J, Liu J et al. Epigenome-based splicing prediction using a recurrent neural network. PLoS Comput Biol. 2020;16:e1008006. 10.1371/journal.pcbi.1008006. 32584815 PMC7343189

[18] Agirre E, Oldfield A, Bellora N et al. Splicing-associated chromatin signatures: a combinatorial and position-dependent role for histone marks in splicing definition. Nat Commun. 2021;12:682. 10.1038/s41467-021-20979-x. 33514745 PMC7846797

[19] Manz Q, List M. Revisiting evidence for epigenetic control of alternative splicing. bioRxiv, 10.1101/2024.08.30.610315, 1 September 2024, preprint: not peer reviewed.

[20] Do HTT, Shanak S, Barghash A et al. Differential exon usage of developmental genes is associated with deregulated epigenetic marks. Sci Rep. 2023;13:12256. 10.1038/s41598-023-38879-z. 37507411 PMC10382575

[21] Xu Y, Zhao W, Olson SD et al. Alternative splicing links histone modifications to stem cell fate decision. Genome Biol. 2018;19:1–21. 10.1186/s13059-018-1512-3. 30217220 PMC6138936

[22] Hu Q, Greene CS, Heller EA. Specific histone modifications associate with alternative exon selection during mammalian development. Nucleic Acids Res. 2020;48:4709–24. 10.1093/nar/gkaa248. 32319526 PMC7229819

[23] Quinlan AR, Hall IM. BEDTools: a flexible suite of utilities for comparing genomic features. Bioinformatics. 2010;26:841–2. 10.1093/bioinformatics/btq033. 20110278 PMC2832824

[24] Shen S, Park JW, Lu Zx et al. rMATS: robust and flexible detection of differential alternative splicing from replicate RNA-Seq data. Proc Natl Acad Sci. 2014;111:E5593–601. 10.1073/pnas.1419161111. 25480548 PMC4280593

[25] Shao Z, Zhang Y, Yuan GC et al. MAnorm: a robust model for quantitative comparison of ChIP-Seq data sets. Genome Biol. 2012;13:1–17. 10.1186/gb-2012-13-3-r16. PMC3439967 22424423

[26] Yeo G, Burge CB. Maximum entropy modeling of short sequence motifs with applications to RNA splicing signals. In: J Comput Biol. 2004;11:377–94. 10.1089/1066527041410418. 15285897

[27] Paz I, Kosti I, Ares M Jr et al. RBPmap: a web server for mapping binding sites of RNA-binding proteins. Nucleic Acids Res. 2014;42:W361–7. 10.1093/nar/gku406. 24829458 PMC4086114

[28] Ray D, Kazan H, Cook KB et al. A compendium of RNA-binding motifs for decoding gene regulation. Nature. 2013;499:172–7. 10.1038/nature12311. 23846655 PMC3929597

[29] Yamada K, Hamada M. Prediction of RNA–protein interactions using a nucleotide language model. Bioinform Adv. 2022;2:vbac023. 10.1093/bioadv/vbac023. 36699410 PMC9710633

[30] Tareen A, Kinney JB. Logomaker: beautiful sequence logos in Python. Bioinformatics. 2020;36:2272–4. 10.1093/bioinformatics/btz921. 31821414 PMC7141850

[31] Pedregosa F, Varoquaux G, Gramfort A et al. Scikit-learn: Machine Learning in Python. J Mach Learn Res. 2011;12:2825–30.

[32] Hahne F, Ivanek R. Visualizing genomic data using Gviz and bioconductor. Methods Mol Biol. 2016;1418:335–51. 10.1007/978-1-4939-3578-9_16. 27008022

[33] Ramírez F, Dündar F, Diehl S et al. deepTools: a flexible platform for exploring deep-sequencing data. Nucleic Acids Res. 2014;42:W187–91. 10.1093/nar/gku365. 24799436 PMC4086134

[34] Liao Y, Smyth GK, Shi W. featureCounts: an efficient general purpose program for assigning sequence reads to genomic features. Bioinformatics. 2014;30:923–30. 10.1093/bioinformatics/btt656. 24227677

[35] Robinson MD, McCarthy DJ, Smyth GK. edgeR: a Bioconductor package for differential expression analysis of digital gene expression data. bioinformatics. 2010;26:139–40. 10.1093/bioinformatics/btp616. 19910308 PMC2796818

[36] Marakulina D, Vorontsov IE, Kulakovskiy IV et al. EpiFactors 2022: expansion and enhancement of a curated database of human epigenetic factors and complexes. Nucleic Acids Res. 2023;51:D564–70. 10.1093/nar/gkac989. 36350659 PMC9825597

[37] Szklarczyk D, Kirsch R, Koutrouli M et al. The STRING database in 2023: protein–protein association networks and functional enrichment analyses for any sequenced genome of interest. Nucleic Acids Res. 2023;51:D638–46. 10.1093/nar/gkac1000. 36370105 PMC9825434

[38] Gerstberger S, Hafner M, Tuschl T. A census of human RNA-binding proteins. Nat Rev Genet. 2014;15:829–45. 10.1038/nrg3813. 25365966 PMC11148870

[39] Toloşi L, Lengauer T. Classification with correlated features: unreliability of feature ranking and solutions. Bioinformatics. 2011;27:1986–94. 10.1093/bioinformatics/btr300. 21576180

[40] Wang M, Marin A. Characterization and prediction of alternative splice sites. Gene. 2006;366:219–27. 10.1016/j.gene.2005.07.015. 16226402

[41] Cui Y, Cai M, Stanley HE. Comparative analysis and classification of cassette exons and constitutive exons. BioMed Res Int. 2017;2017:7323508. 10.1155/2017/7323508. 29349080 PMC5734011

[42] Shao C, Yang B, Wu T et al. Mechanisms for U2AF to define 3′ splice sites and regulate alternative splicing in the human genome. Nat Struct Mol Biol. 2014;21:997–1005. 10.1038/nsmb.2906. 25326705 PMC4429597

[43] Sims RJ, Millhouse S, Chen CF et al. Recognition of trimethylated histone H3 lysine 4 facilitates the recruitment of transcription postinitiation factors and pre-mRNA splicing. Mol Cell. 2007;28:665–76. 10.1016/j.molcel.2007.11.010. 18042460 PMC2276655

[44] Wu W, Ahmad K, Henikoff S. Chromatin-bound U2AF2 splicing factor ensures exon inclusion. Mol Cell. 2025;85:1982–98. 10.1016/j.molcel.2025.04.013. 40315850 PMC13075997

[45] Barreau C, Paillard L, Osborne HB. AU-rich elements and associated factors: are there unifying principles?. Nucleic Acids Res. 2005;33:7138–50. 10.1093/nar/gki1012. 16391004 PMC1325018

[46] Huang H, Weng H, Chen J. The biogenesis and precise control of RNA m6A methylation. Trends Genet. 2020;36:44–52. 10.1016/j.tig.2019.10.011. 31810533 PMC6925345

[47] Wilson C, Kanhere A. Investigating the role of CpG island DNA methylation at 3’UTRs in cancer. bioRxiv, 10.1101/2024.10.18.619008, 21 October 2024, preprint: not peer reviewed. PMC13401056 42498499

[48] Bakheet T, Hitti E, Al-Saif M et al. The AU-rich element landscape across human transcriptome reveals a large proportion in introns and regulation by ELAVL1/HuR. Biochimica et Biophysica Acta (BBA)-Gene Regulatory Mechanisms. 2018;1861:167–77. 10.1016/j.bbagrm.2017.12.006. 29413897

[49] Gutierrez-Arcelus M, Ongen H, Lappalainen T et al. Tissue-specific effects of genetic and epigenetic variation on gene regulation and splicing. PLoS Genet. 2015;11:e1004958. 10.1371/journal.pgen.1004958. 25634236 PMC4310612

[50] Koch CM, Andrews RM, Flicek P et al. The landscape of histone modifications across 1% of the human genome in five human cell lines. Genome Res. 2007;17:691–707. 10.1101/gr.5704207. 17567990 PMC1891331

[51] Cardone J, Le Friec G, Kemper C. CD46 in innate and adaptive immunity: an update. Clin Exp Immunol. 2011;164:301–11. 10.1111/j.1365-2249.2011.04400.x. 21488871 PMC3087925

[52] Hirano A, Yang Z, Katayama Y et al. Human CD46 enhances nitric oxide production in mouse macrophages in response to measles virus infection in the presence of gamma interferon: dependence on the CD46 cytoplasmic domains. J Virol. 1999;73:4776–85. 10.1128/JVI.73.6.4776-4785.1999. 10233938 PMC112520

[53] Wang G, Liszewski MK, Chan AC et al. Membrane cofactor protein (MCP; CD46): isoform-specific tyrosine phosphorylation. J Immunol. 2000;164:1839–46. 10.4049/jimmunol.164.4.1839. 10657632

[54] Förch P, Puig O, Kedersha N et al. The apoptosis-promoting factor TIA-1 is a regulator of alternative pre-mRNA splicing. Mol Cell. 2000;6:1089–98. 10.1016/S1097-2765(00)00107-6. 11106748

[55] Del Gatto-Konczak F, Bourgeois CF, Le Guiner C et al. The RNA-binding protein TIA-1 is a novel mammalian splicing regulator acting through intron sequences adjacent to a 5′ splice site. Mol Cell Biol. 2000;20:6287–99. 10.1128/MCB.20.17.6287-6299.2000. 10938105 PMC86103

[56] Förch P, Puig O, Martínez C et al. The splicing regulator TIA-1 interacts with U1-C to promote U1 snRNP recruitment to 5′ splice sites. EMBO J. 2002;21:6882–92. 10.1093/emboj/cdf668. 12486009 PMC139089

[57] Tang SJ, Luo S, Ho JXJ et al. Characterization of the regulation of CD46 RNA alternative splicing. J Biol Chem. 2016;291:14311–23. 10.1074/jbc.M115.710350. 27226545 PMC4933185

[58] Wang Z, Kayikci M, Briese M et al. iCLIP predicts the dual splicing effects of TIA-RNA interactions. PLoS Biol. 2010;8:e1000530. 10.1371/journal.pbio.1000530. 21048981 PMC2964331

[59] Jostins L, Ripke S, Weersma RK et al. Host–microbe interactions have shaped the genetic architecture of inflammatory bowel disease. Nature. 2012;491:119–24. 10.1038/nature11582. 23128233 PMC3491803

[60] Sandhu G, Thelma B. New druggable targets for rheumatoid arthritis based on insights from synovial biology. Front Immunol. 2022;13:834247. 10.3389/fimmu.2022.834247. 35265082 PMC8899708

[61] Croft M, Benedict CA, Ware CF. Clinical targeting of the TNF and TNFR superfamilies. Nat Rev Drug Discov. 2013;12:147–68. 10.1038/nrd3930. 23334208 PMC3625401

[62] Sobajima T, Aoki F, Kohmoto K. Activation of mitogen-activated protein kinase during meiotic maturation in mouse oocytes. Reproduction. 1993;97:389–94. 10.1530/jrf.0.0970389. 7684786

[63] Meyer C, Garzia A, Mazzola M et al. The TIA1 RNA-binding protein family regulates EIF2AK2-mediated stress response and cell cycle progression. Mol Cell. 2018;69:622–35. 10.1016/j.molcel.2018.01.011. 29429924 PMC5816707

[64] Cho S, Moon H, Loh TJ et al. Splicing inhibition of U2AF65 leads to alternative exon skipping. Proc Natl Acad Sci. 2015;112:9926–31. 10.1073/pnas.1500639112. 26216990 PMC4538632

[65] Genuer R, Poggi JM, Tuleau C. Random Forests: some methodological insights. arXiv preprint arXiv:08113619, 2008.

[66] Fu XD, Ares M Jr. Context-dependent control of alternative splicing by RNA-binding proteins. Nat Rev Genet. 2014;15:689–701. 10.1038/nrg3778. 25112293 PMC4440546

[67] Zhou HL, Luo G, Wise JA et al. Regulation of alternative splicing by local histone modifications: potential roles for RNA-guided mechanisms. Nucleic Acids Res. 2013;42:701–13. 10.1093/nar/gkt875. 24081581 PMC3902899

[68] Sengupta D, Kannan A, Kern M et al. Disruption of BRD4 at H3K27Ac-enriched enhancer region correlates with decreased c-Myc expression in Merkel cell carcinoma. Epigenetics. 2015;10:460–6. 10.1080/15592294.2015.1034416. 25941994 PMC4622756

[69] Quinones-Valdez G, Tran SS, Jun HI et al. Regulation of RNA editing by RNA-binding proteins in human cells. Commun Biol. 2019;2:19. 10.1038/s42003-018-0271-8. 30652130 PMC6331435

[70] Shadrina O, Garanina I, Korolev S et al. Analysis of RNA binding properties of human Ku protein reveals its interactions with 7SK snRNA and protein components of 7SK snRNP complex. Biochimie. 2020;171:110–23. 10.1016/j.biochi.2020.02.016. 32105815

[71] Jin Q, Yu LR, Wang L et al. Distinct roles of GCN5/PCAF-mediated H3K9ac and CBP/p300-mediated H3K18/27ac in nuclear receptor transactivation. EMBO J. 2011;30:249–62. 10.1038/emboj.2010.318. 21131905 PMC3025463

[72] Liang Z, Rehati A, Husaiyin E et al. RALY regulate the proliferation and expression of immune/inflammatory response genes via alternative splicing of FOS. Genes Immun. 2022;23:246–54. 10.1038/s41435-022-00178-4. 35941292 PMC9758052

[73] Liang Z, Rehati A, Husaiyin E et al. RALY regulate the proliferation and expression of immune/inflammatory response genes via alternative splicing of FOS. Genes Immun. 2022;23:246–54. 10.1038/s41435-022-00178-4. 35941292 PMC9758052

[74] Wu H, Zeng H, Lam R et al. Structural and histone binding ability characterizations of human PWWP domains. PloS One. 2011;6:e18919. 10.1371/journal.pone.0018919. 21720545 PMC3119473

[75] Schmok JC, Jain M, Street LA et al. Large-scale evaluation of the ability of RNA-binding proteins to activate exon inclusion. Nat Biotechnol. 2024;42:1429–41. 38168984 10.1038/s41587-023-02014-0 PMC11389820

[76] Sun XJ, Wei J, Wu XY et al. Identification and characterization of a novel human histone H3 lysine 36-specific methyltransferase. J Biol Chem. 2005;280:35261–71. 10.1074/jbc.M504012200. 16118227

[77] Kizer KO, Phatnani HP, Shibata Y et al. A novel domain in Set2 mediates RNA polymerase II interaction and couples histone H3 K36 methylation with transcript elongation. Mol Cell Biol. 2005;25:3305–16. 10.1128/MCB.25.8.3305-3316.2005. 15798214 PMC1069628

[78] De Almeida SF, Grosso AR, Koch F et al. Splicing enhances recruitment of methyltransferase HYPB/Setd2 and methylation of histone H3 Lys36. Nat Struct Mol Biol. 2011;18:977–83. 10.1038/nsmb.2123. 21792193

[79] Strikoudis A, Lazaris C, Trimarchi T et al. Regulation of transcriptional elongation in pluripotency and cell differentiation by the PHD-finger protein Phf5a. Nat Cell Biol. 2016;18:1127–38. 10.1038/ncb3424. 27749823 PMC5083132

[80] Caron P, van Der Linden J, van Attikum H. Bon voyage: a transcriptional journey around DNA breaks. DNA Repair. 2019;82:102686. 10.1016/j.dnarep.2019.102686. 31476573

[81] Wolf D, Goff SP. TRIM28 mediates primer binding site-targeted silencing of murine leukemia virus in embryonic cells. Cell. 2007;131:46–57. 10.1016/j.cell.2007.07.026. 17923087

[82] Wang B, Li L, Zhu Y et al. Sequence variants of KHDRBS1 as high penetrance susceptibility risks for primary ovarian insufficiency by mis-regulating mRNA alternative splicing. Hum Reprod. 2017;32:2138–46. 10.1093/humrep/dex263. 28938739

[83] Yearim A, Gelfman S, Shayevitch R et al. HP1 is involved in regulating the global impact of DNA methylation on alternative splicing. Cell Rep. 2015;10:1122–34. 10.1016/j.celrep.2015.01.038. 25704815

[84] Ustianenko D, Weyn-Vanhentenryck SM, Zhang C. Microexons: discovery, regulation, and function. Wiley Interdiscip Rev RNA. 2017;8:e1418. 10.1002/wrna.1418. PMC5863539 28188674

[85] Small SJ, Haines SL, Akeson RA. Polypeptide variation in an N-CAM extracellular immunoglobulin-like fold is developmentally regulated through alternative splicing. Neuron. 1988;1:1007–17. 10.1016/0896-6273(88)90158-4. 2483093

[86] Irimia M, Weatheritt RJ, Ellis JD et al. A highly conserved program of neuronal microexons is misregulated in autistic brains. Cell. 2014;159:1511–23. 10.1016/j.cell.2014.11.035. 25525873 PMC4390143

[87] Lee JS, Lamarche-Vane N, Richard S. Microexon alternative splicing of small GTPase regulators: Implication in central nervous system diseases. Wiley Interdiscip Rev RNA. 2022;13:e1678. 10.1002/wrna.1678. 34155820

[88] Carlo T, Sierra R, Berget SM. A 5′ splice site-proximal enhancer binds SF1 and activates exon bridging of a microexon. Mol Cell Biol. 2000;20:3988–95. 10.1128/MCB.20.11.3988-3995.2000. 10805741 PMC85762

[89] Li YI, Sanchez-Pulido L, Haerty W et al. RBFOX and PTBP1 proteins regulate the alternative splicing of micro-exons in human brain transcripts. Genome Res. 2015;25:1–13. 10.1101/gr.181990.114. 25524026 PMC4317164

[90] Uhl M, Tran VD, Heyl F et al. RNAProt: an efficient and feature-rich RNA binding protein binding site predictor. GigaScience. 2021;10:giab054. 10.1093/gigascience/giab054. 34406415 PMC8372218

[91] Pan X, Rijnbeek P, Yan J et al. Prediction of RNA-protein sequence and structure binding preferences using deep convolutional and recurrent neural networks. BMC Genomics. 2018;19:511. 10.1186/s12864-018-4889-1. 29970003 PMC6029131

[92] Maticzka D, Lange SJ, Costa F et al. GraphProt: modeling binding preferences of RNA-binding proteins. Genome Biol. 2014;15:R17. 10.1186/gb-2014-15-1-r17. 24451197 PMC4053806

[93] Xu Y, Zhu J, Huang W et al. PrismNet: predicting protein–RNA interaction using in vivo RNA structural information. Nucleic Acids Res. 2023;51:W468–77. 10.1093/nar/gkad353. 37140045 PMC10320048

