# Supplementary material for: RNA-binding proteins connect Exon usage to the chromatin
Source: NAR Genom Bioinform. 2025 Dec 10;7(4):lqaf161. doi: 10.1093/nargab/lqaf161 (PMC12693533; doi:10.1093/nargab/lqaf161)
Supplement: lqaf161_Supplemental_Files [file lqaf161_supplemental_files.zip › Supplementary_Figures__Revision_.pdf]

Supplementary Figures

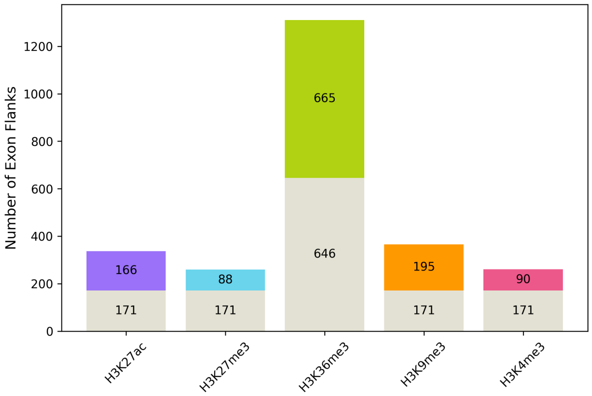

(A) Number of epispliced and non-epispliced exon flanks

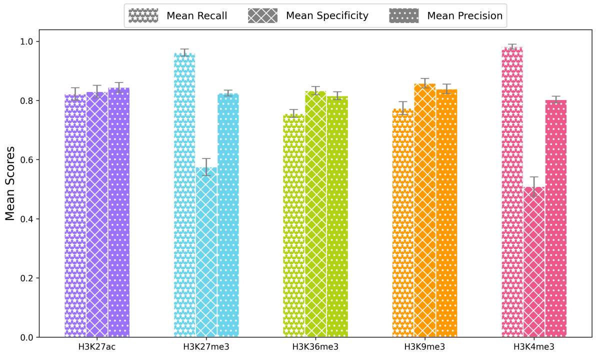

(B) Performance metrics of the binary classifiers

Figure S1. **(A)** Number of epispliced exon flanks (±200bp) after pooling them for each histone mark. Number of non-epsipliced exon flanks (grey) reported in at least 70% of the analyses were compiled as a common control set. **(B)** Performance metrics of the binary classifiers trained using binding affinities of RNA-binding protein (RBP) on the flanking regions of exons that are differentially spliced among embryonic cell lines.

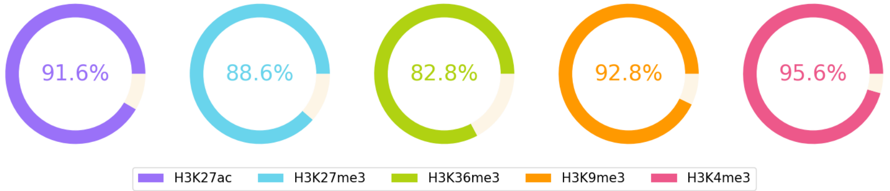

Figure S2. Percentage of internal epispliced exons. Alternate first exons were previously excluded as shown in Figure 2A. Exons occurring within a ±200bp window of the transcription termination sites were extracted as the alternate last exons. Most epispliced exons were found outside this window, signifying internal exons.

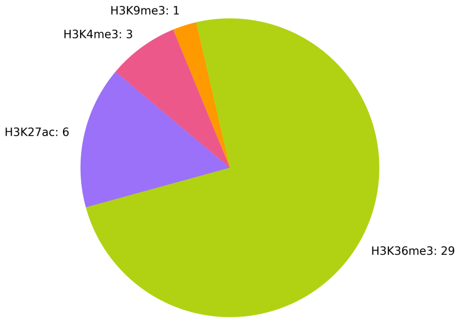

(A) Number of epispliced genes in the cancer cell lines.

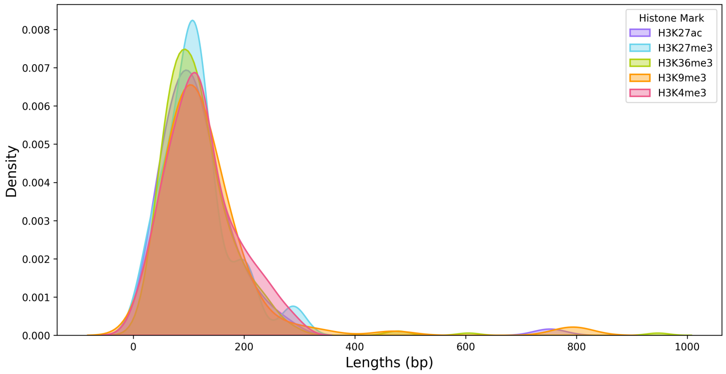

(B) Lengths of epispliced exons

Figure S3. **(A)** Number of epispliced genes identified in the HepG2-K562 cell lines. **(B)** Length distribution of the epispliced exons obtained from the embryonic cell lines (Figure S1A). The majority of epispliced exons were 125-140 base pairs in length. H3K9me3 and H3K36me3 signals were annotated to a few longer exons.

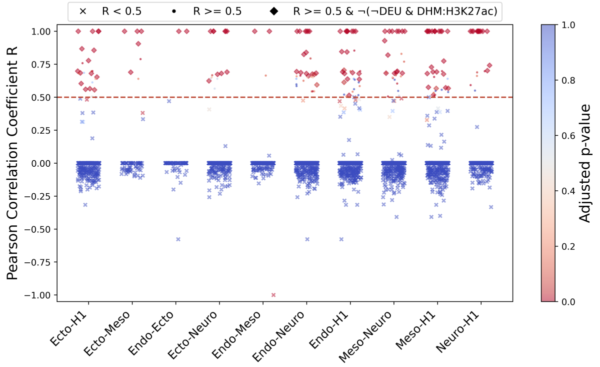

(A) H3K27ac

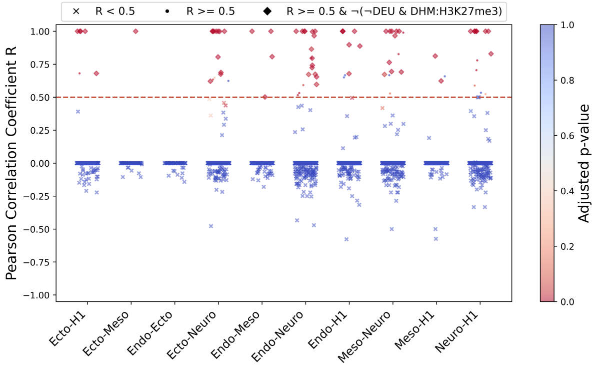

(B) H3K27me3

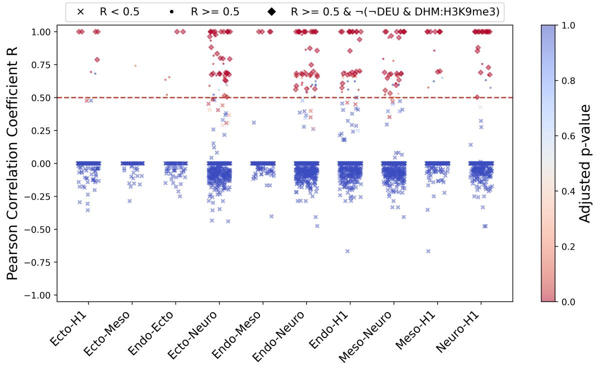

(C) H3K9me3

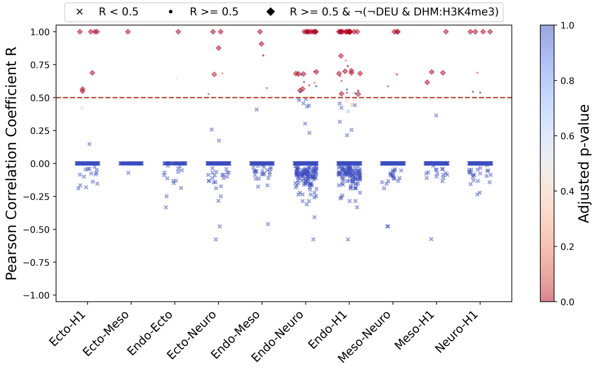

(D) H3K4me3

Figure S4. Genes whose alternative splicing events were correlated to histone modifications annotated only to alternative exons were called *epispliced genes* ($R \geq 0.5$, $p_{FDR} < 0.05$). Manhattan plots illustrating the multistep filtering of candidate genes to obtain epispliced genes associated with H3K27ac, H3K27me3, H3K9me3 and H3K4me3, respectively. Genes passing ($R \geq 0.5$) and failing ($R < 0.5$) the correlation coefficient thresholding are represented using dot and cross markers, respectively. Candidate genes additionally filtered based on alternative exon-specific DHM peak enrichment are represented using diamond markers.

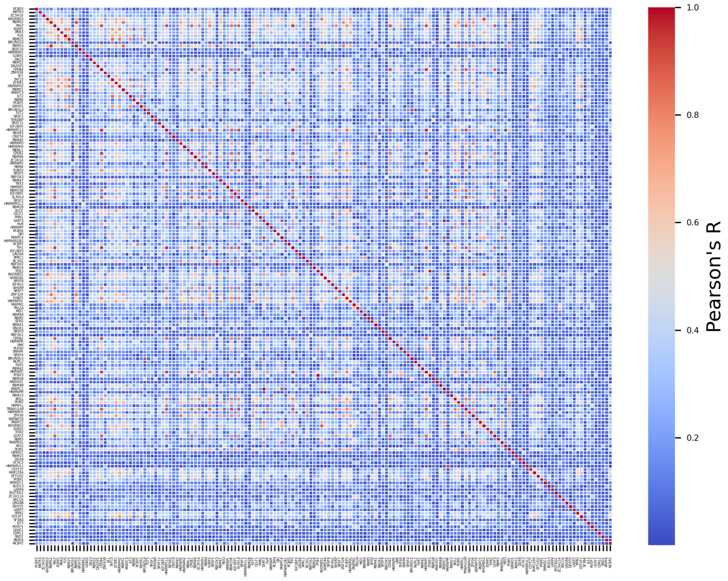

(A) H3K27ac

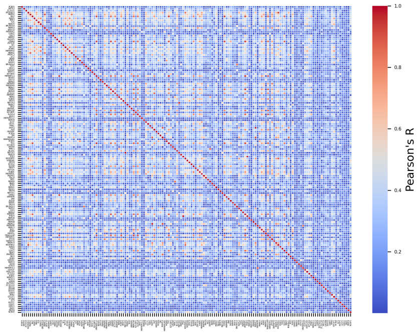

(B) H3K27me3

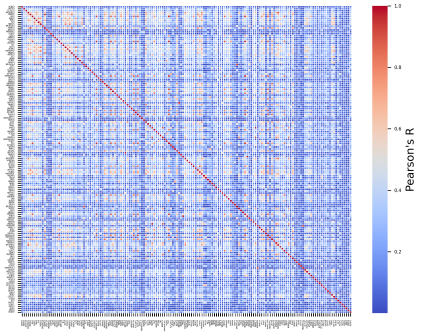

(C) H3K36me3

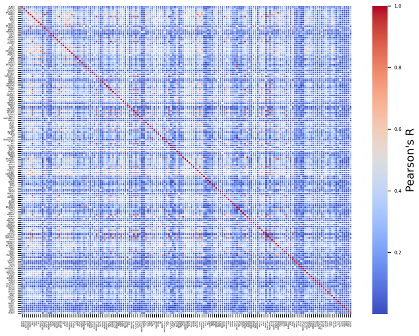

(D) H3K9me3

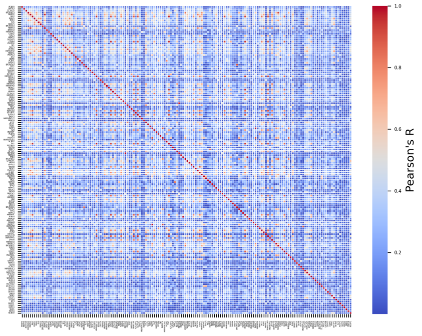

(E) H3K4me3

Figure S5. Pearson correlation matrices of all RBP binding scores for the five histone marks. Shown on the X and Y axes are all considered 160 RBPs.

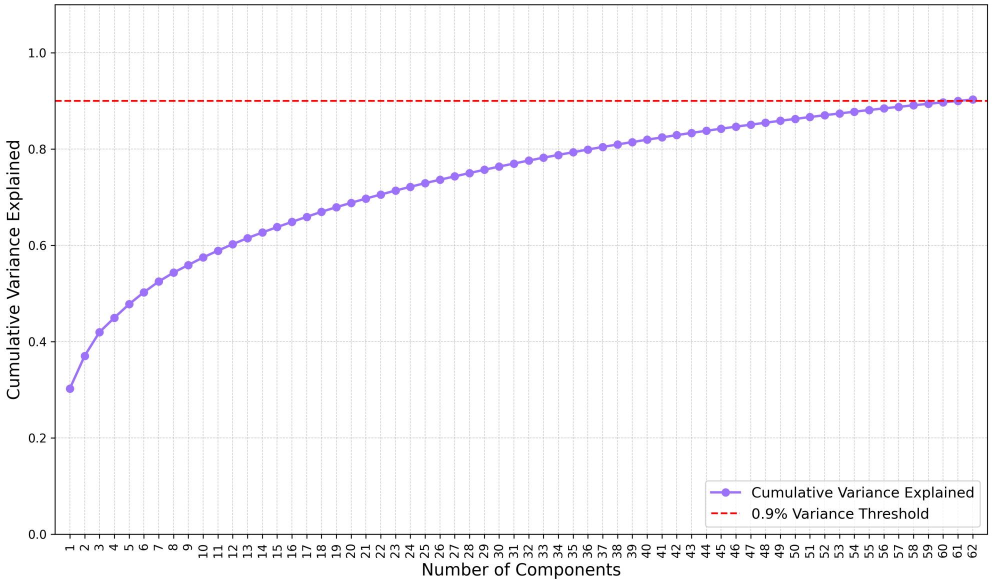

(A) H3K27ac

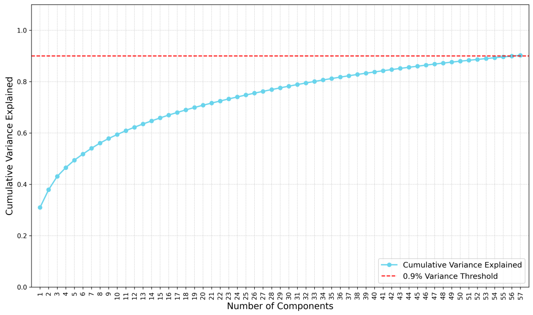

(B) H3K27me3

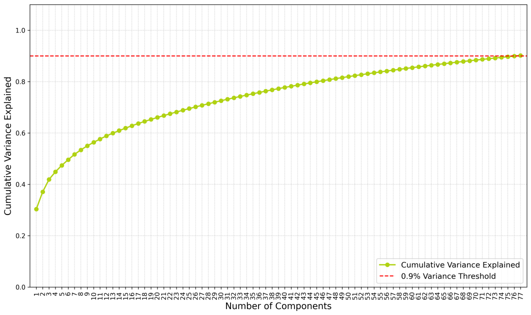

(C) H3K36me3

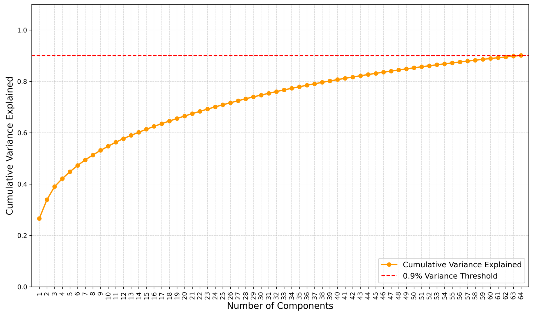

(D) H3K9me3

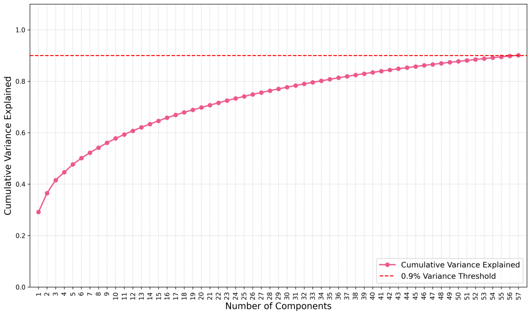

(E) H3K4me3

Figure S6. Cumulative explained variance plot for all five histone mark datasets. The principal components shown on the X-axis reflect different combinations of RBPs (PCA vectors).

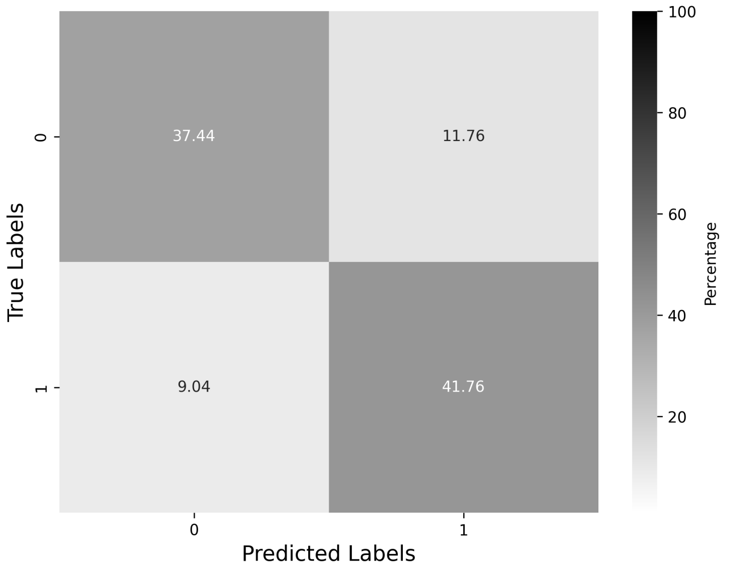

(A) H3K36me3

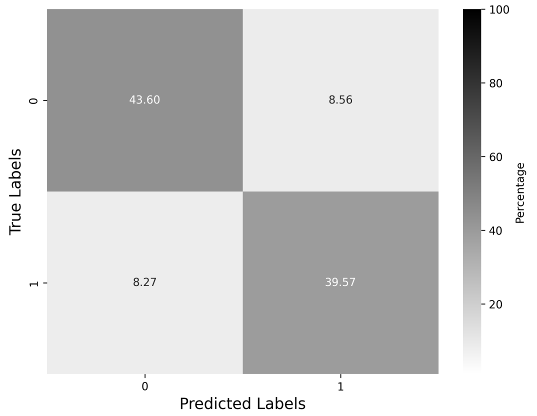

(B) H3K27ac

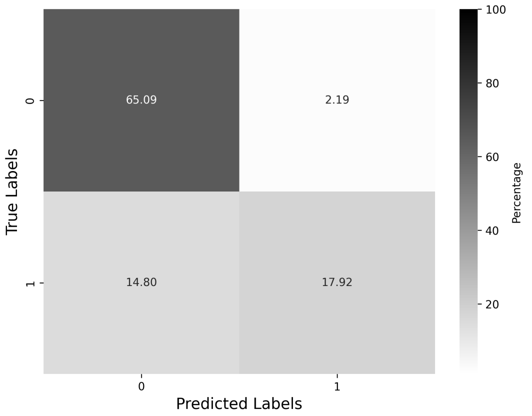

(C) H3K27me3

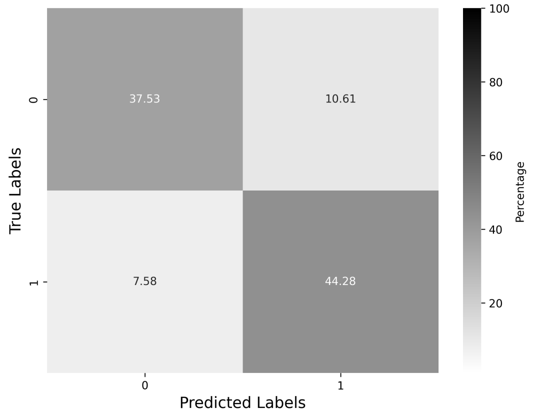

(D) H3K9me3

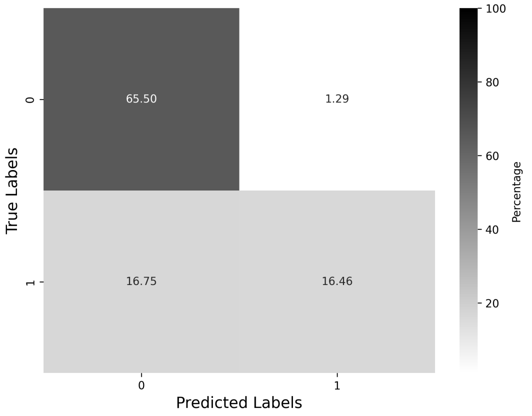

(E) H3K4me3

Figure S7. Confusion matrices of all five models. 1: Epispliced exon flanks, 0: Non-epispliced exon flanks.

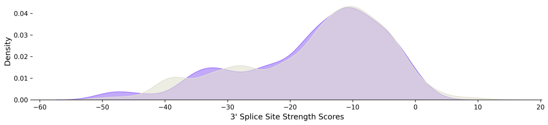

(A) H3K27ac - 3’ Splice Site Strengths

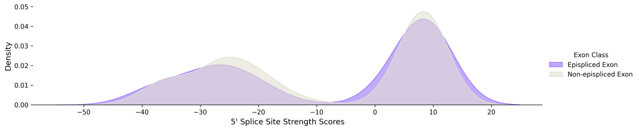

(B) H3K27ac - 5’ Splice Site Strengths

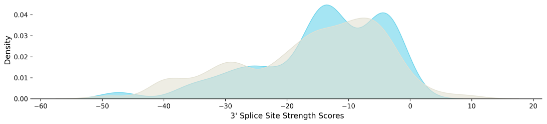

(C) H3K27me3 - 3’ Splice Site Strengths

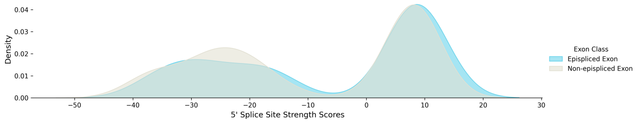

(D) H3K27me3 - 5’ Splice Site Strengths

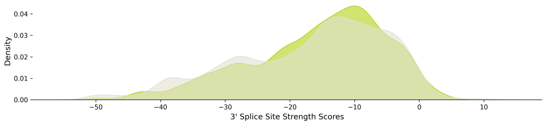

(E) H3K36me3 - 3’ Splice Site Strengths

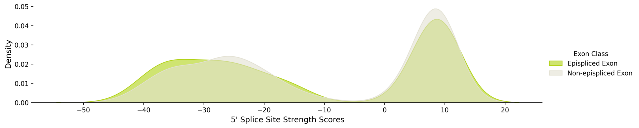

(F) H3K36me3 - 5’ Splice Site Strengths

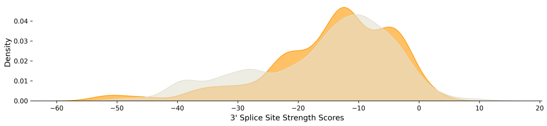

(G) H3K9me3 - 3’ Splice Site Strengths

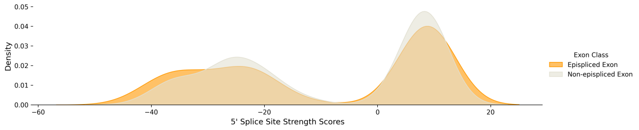

(H) H3K9me3 - 5’ Splice Site Strengths

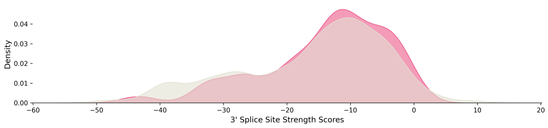

(I) H3K4me3 - 3’ Splice Site Strengths

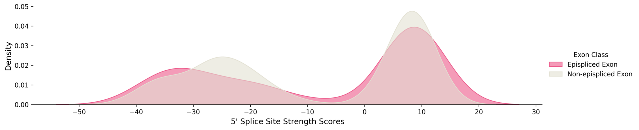

(J) H3K4me3 - 5’ Splice Site Strengths

Figure S8. Ridge plots showing the distribution of the 3’ and 5’ splice site strengths between the epispliced and non-epispliced exons of the five histone marks. The splice site strengths were predicted using *MaxEntScan*.

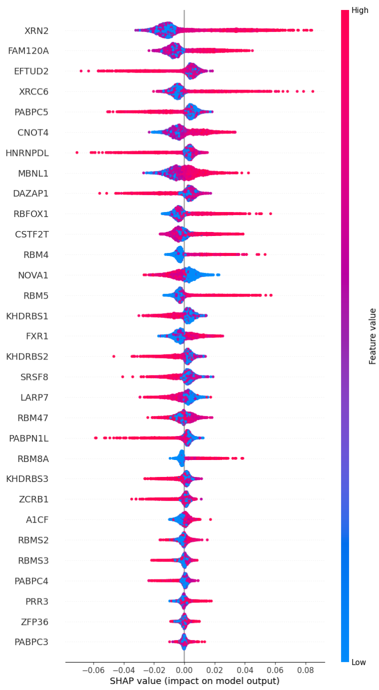

(A) H3K27ac

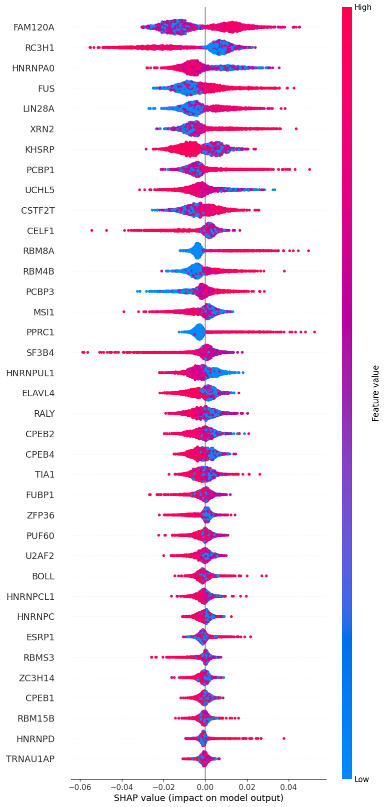

(B) H3K27me3

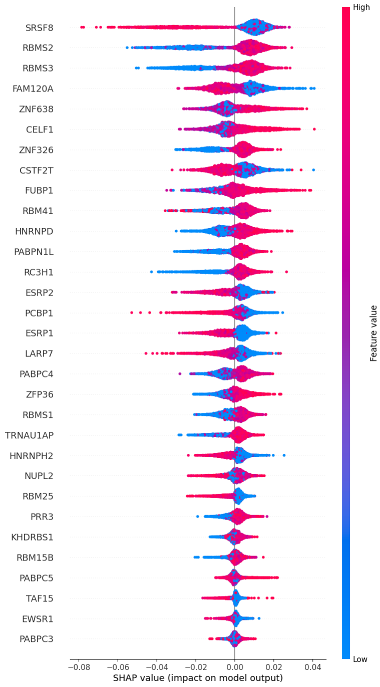

(C) H3K9me3

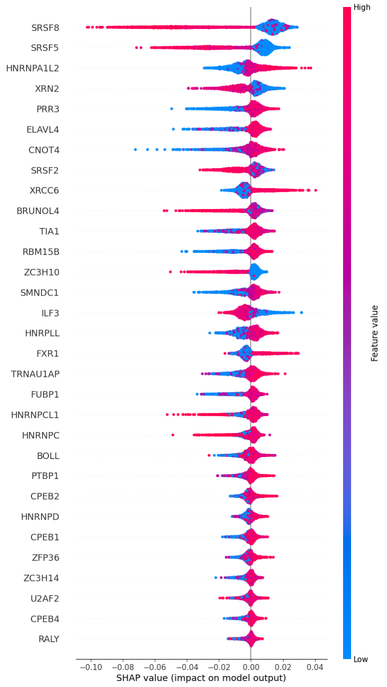

(D) H3K4me3

Figure S9. Mean SHAP values of RBPs were used to ascertain key to model predictors, in order to identify the RBPs that bind preferentially to episliced or non-episliced exon flanks. RBPs whose binding was associated with a strong, positive SHAP value in most flanking regions of epispliced exons were distinguished as episplicing RBPs, while those with marked weak binding in epispliced exon flanks were labeled as non-episplicing RBPs.

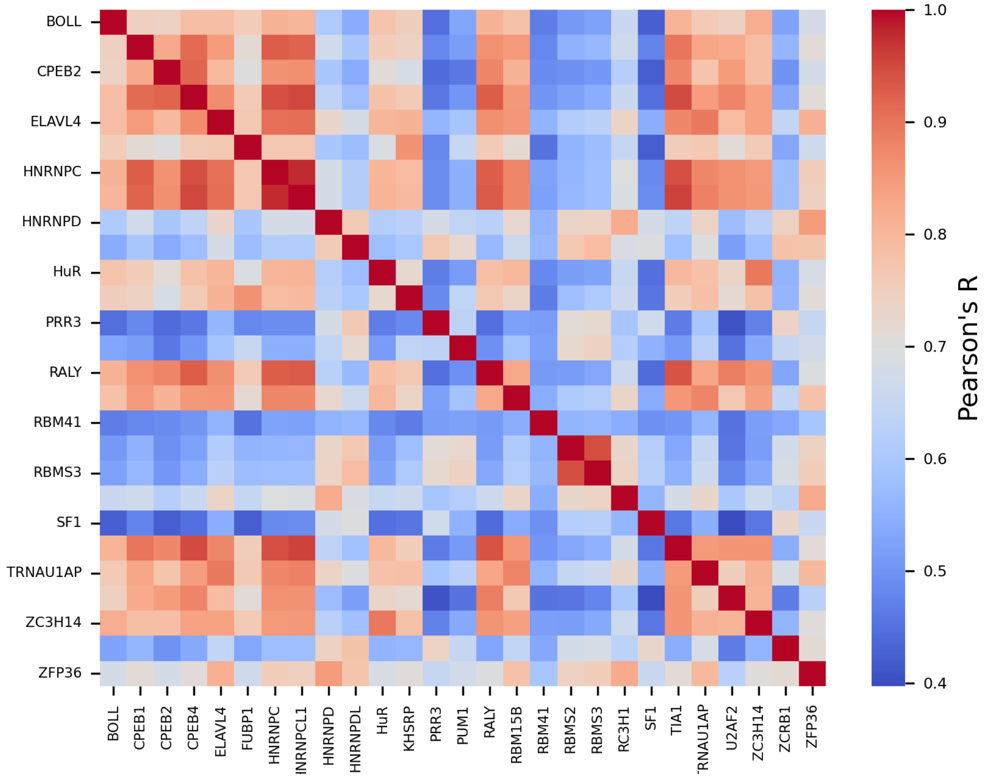

(A) H3K36me3

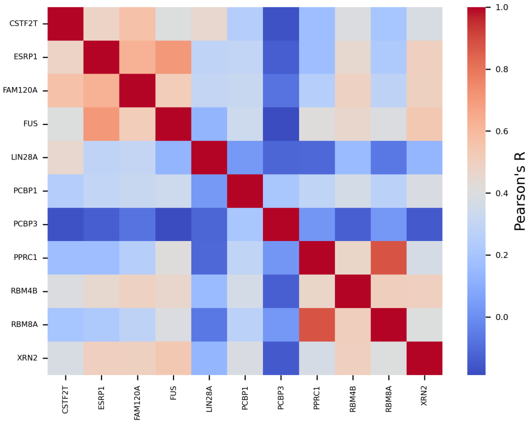

(B) H3K27me3

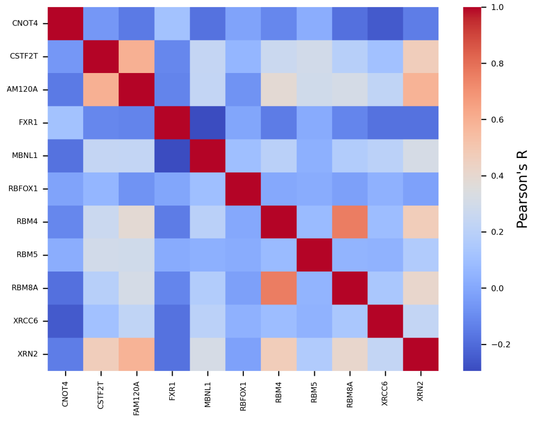

(C) H3K27ac

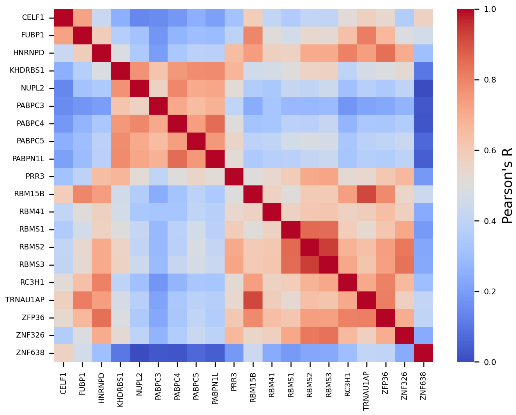

(D) H3K9me3

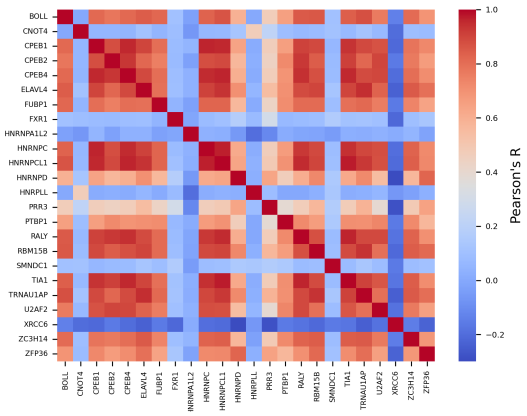

(E) H3K4me3

Figure S10. Correlation matrices of episplicing RBPs predicted for all five histone marks.

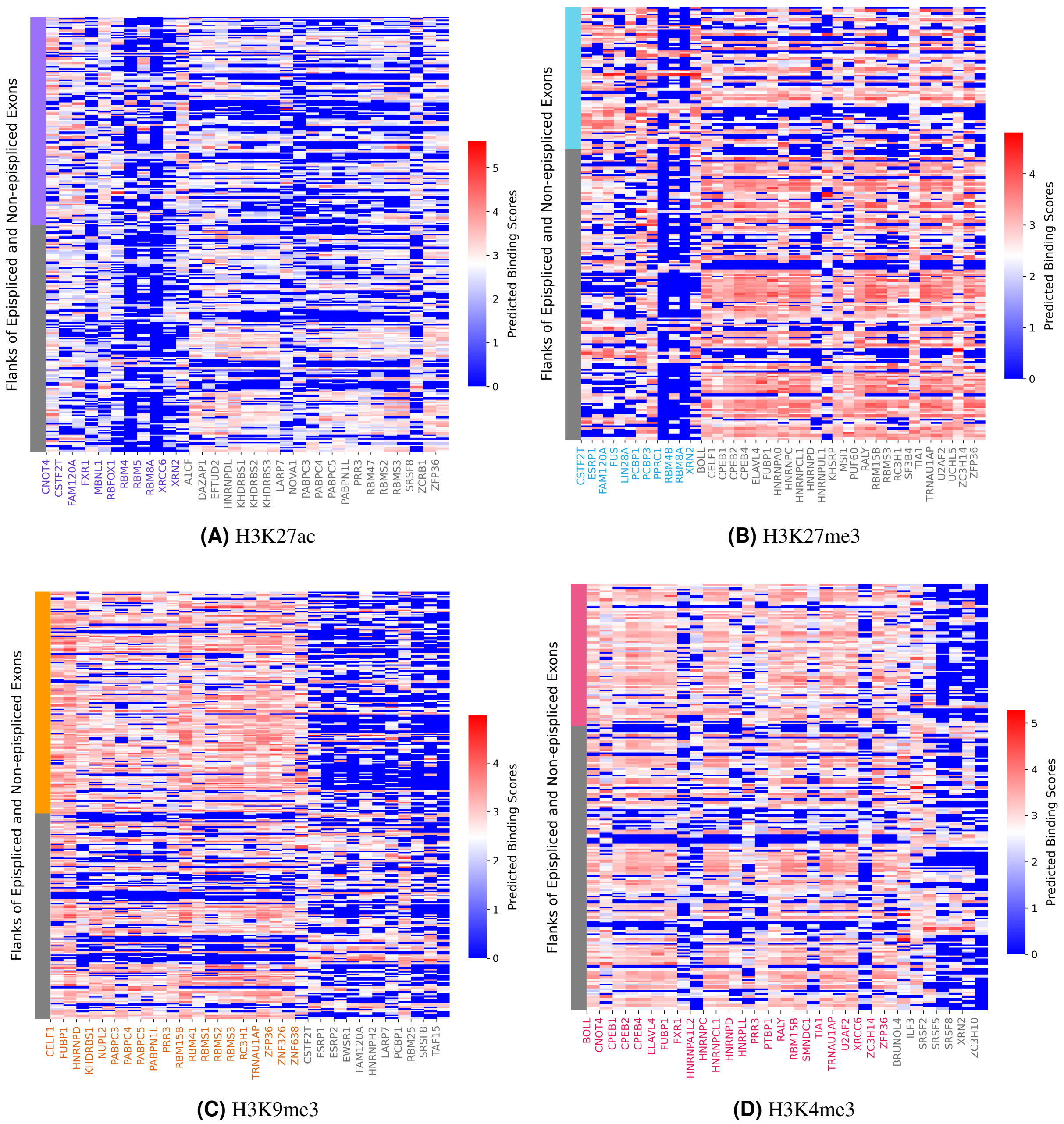

Figure S11. Putative binding scores of the identified episplicing and non-episplicing RBPs associated with H3K27ac, H3K27me3, H3K9me3 and H3K4me3. The flanking sequences of non-epispliced exons are marked by the grey colorbar, while those of epispliced exons are marked in purple, blue, orange and magenta for H3K27ac, H3K27me3, H3K9me3 and H3K4me3, respectively. The episplicing RBPs associated with each mark are highlighted in the same colors.

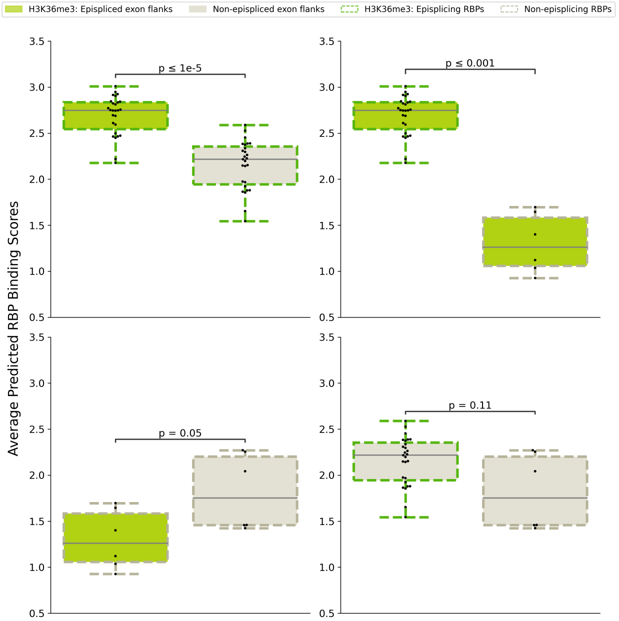

(A) H3K36me3

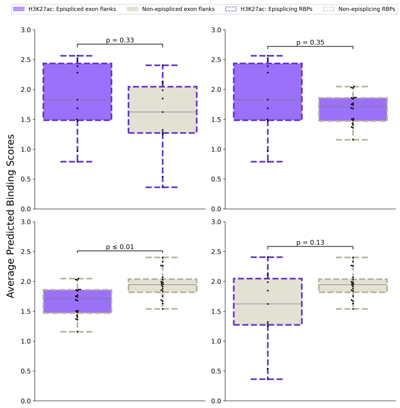

(B) H3K27ac

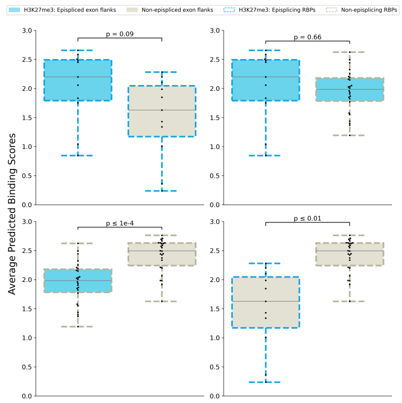

(C) H3K27me3

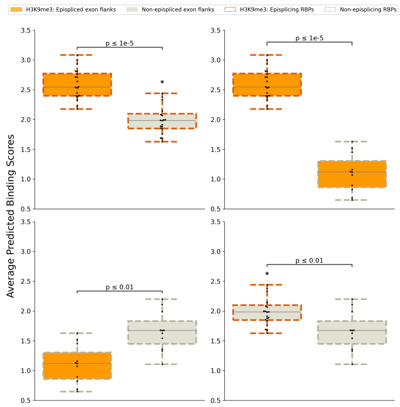

(D) H3K9me3

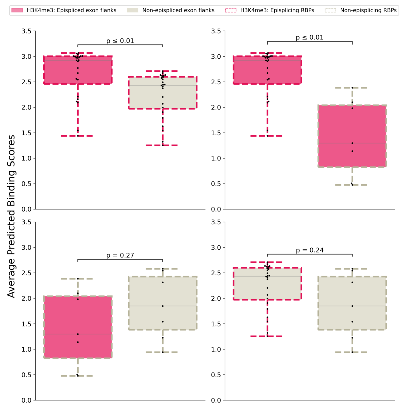

(E) H3K4me3

Figure S12. Welsh's test was employed to compare the binding scores of the episplicing and non-episplicing RBPs obtained from each histone mark model between the flanking sequences of the epispliced and non-epispliced exons. For each mark, the four panels reflect the comparison of the average binding affinities of episplicing RBPs between both classes of exon flanks (upper left), non-episplicing RBPs between both classes of exon flanks (bottom left), both classes of RBPs at the epispliced exon flanks (upper right), and both classes of RBPs at the non-epispliced exon flanks (bottom right), see Figures 5B and S11.

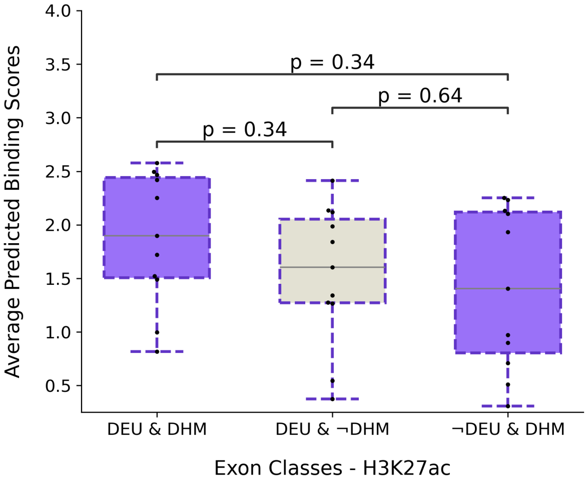

(A) H3K27ac - episplicing RBPs

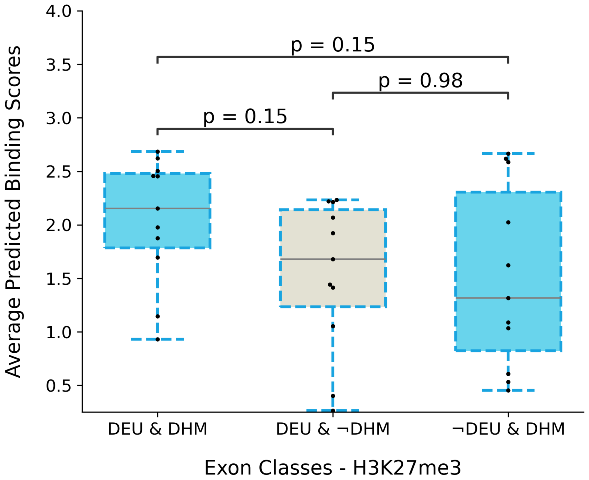

(B) H3K27me3 - episplicing RBPs

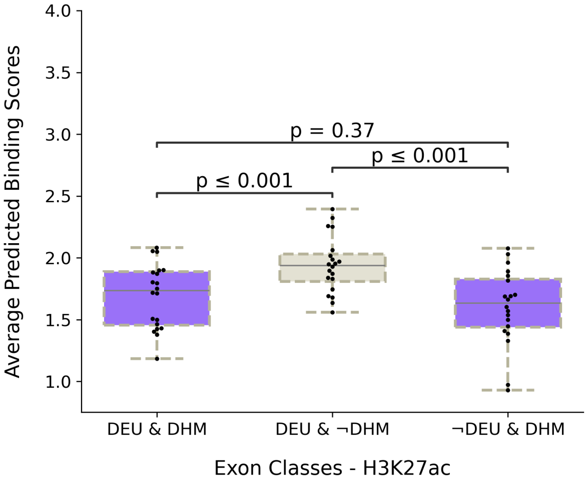

(C) H3K27ac - non-episplicing RBPs

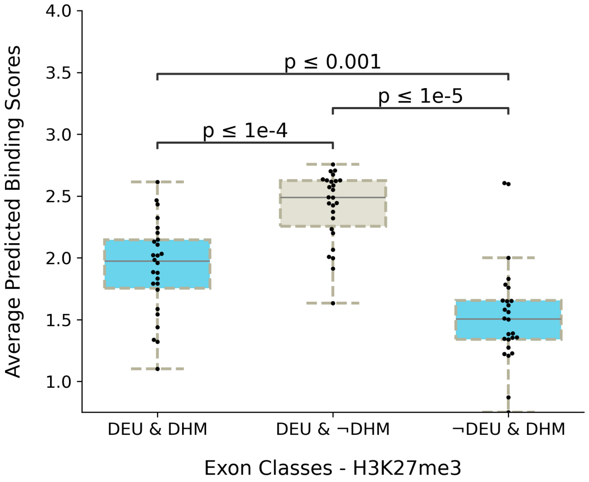

(D) H3K27me3 - non-episplicing RBPs

Figure S13. The distributions of binding affinities of episplicing and non-episplicing RBPs to exon-flanking regions were compared across three classes of exons: epispliced ($DEU\&DHM$), non-epispliced ($DEU\&\neg DHM$) and constitutive exons with deregulated histone signals ($\neg DEU\&DHM$). **(A)**-**(B)** show the binding score distributions of episplicing RBPs predicted to associate with H3K27ac and H3K27me3, respectively. **(C)**-**(D)** show the binding score distributions of non-episplicing RBPs predicted to show weak binding to alternative exons marked by H3K27ac and H3K27me3, respectively.

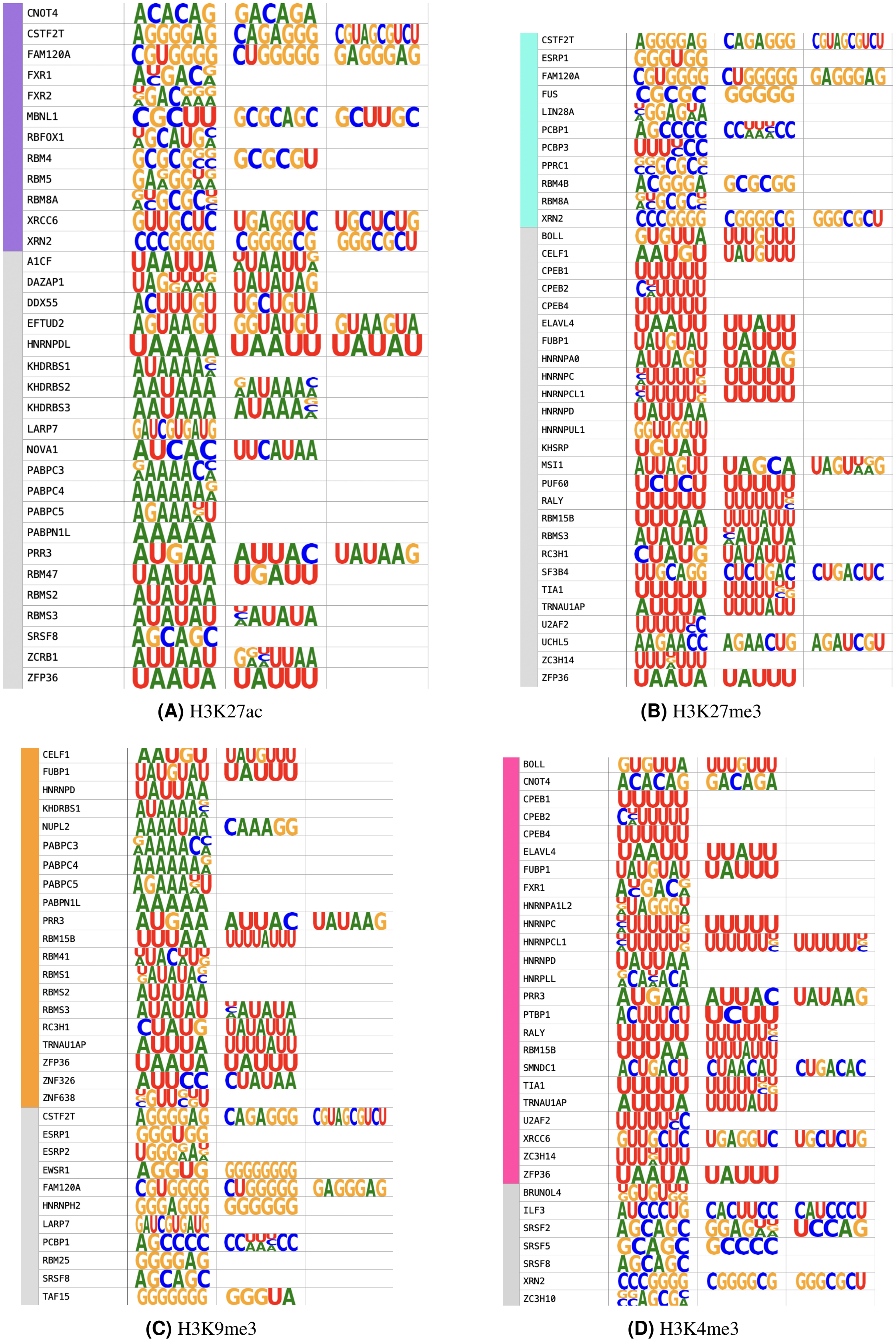

Figure S14. Binding motifs of episplicing and non-episplicing (colored gray) RBPs associated with H3K27ac, H3K27me3, H3K9me3 and H3K4me3, respectively. The episplicing RBPs are marked in purple, blue, orange and magenta for H3K27ac, H3K27me3, H3K9me3 and H3K4me3, respectively.

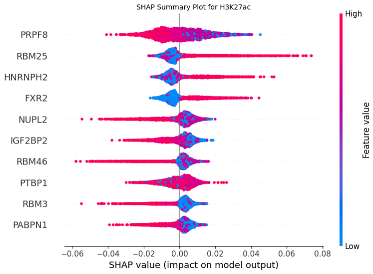

(A) H3K27ac - SHAP

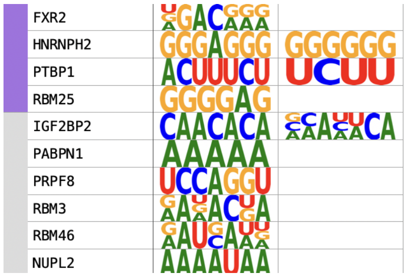

(B) H3K27ac - Motifs

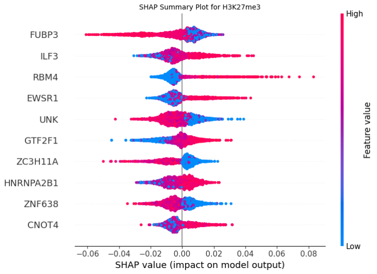

(C) H3K27me3 - SHAP

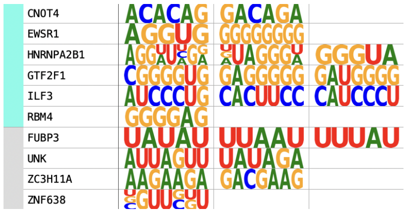

(D) H3K27me3 - Motifs

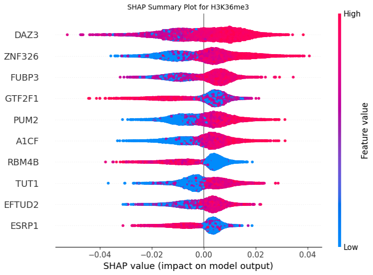

(E) H3K36me3 - SHAP

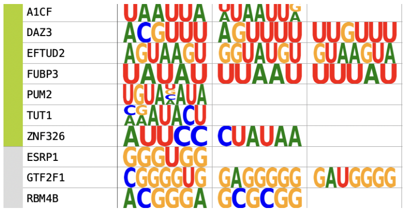

(F) H3K36me3 - Motifs

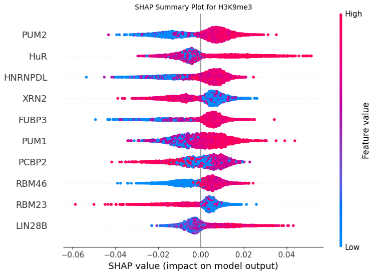

(G) H3K9me3 - SHAP

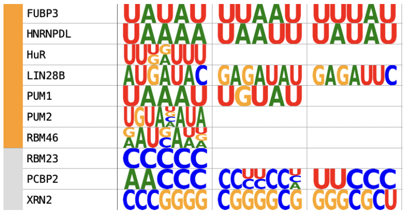

(H) H3K9me3 - Motifs

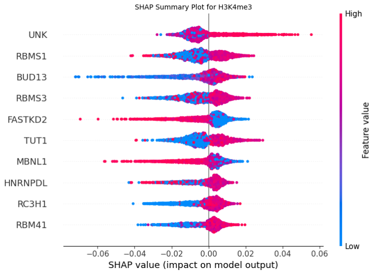

(I) H3K4me3 - SHAP

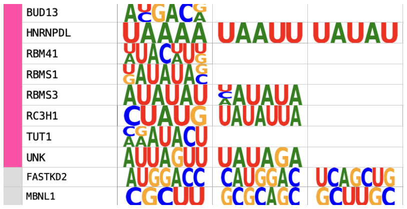

(J) H3K4me3 - Motifs

Figure S15. SHAP summary plots and motifs of the top 10 most important RBPs of the classifiers run after removing the binding scores of episplicing and non-episplicing RBPs (Figures 6B, S14, Supplementary Tables 3,4) reported by the original model.

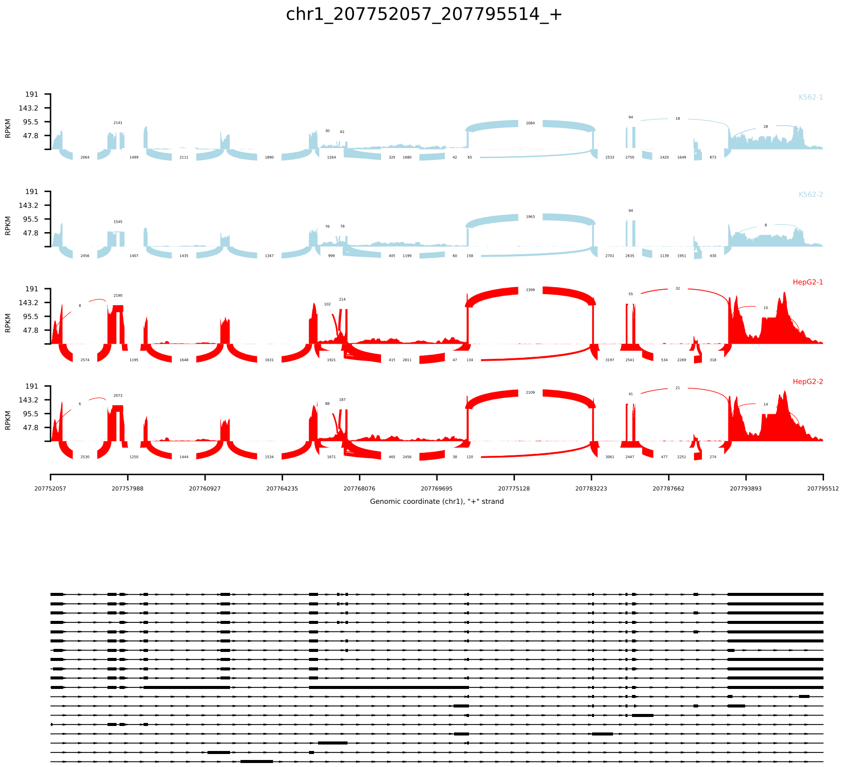

(A) CD46

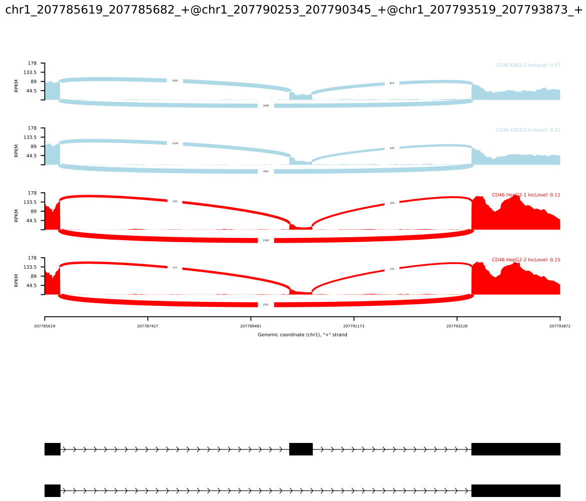

(B) Exon 13 of CD46

Figure S16. **A** Sashimi plot showing the RNA-seq read support for the exon usage events of gene CD46. **B** Sashimi plot showing the RNA-Seq read support for the alternative exon of CD46 marked by H3K36me3 and bound by TIA1 and U2AF2, see Figure 9A.

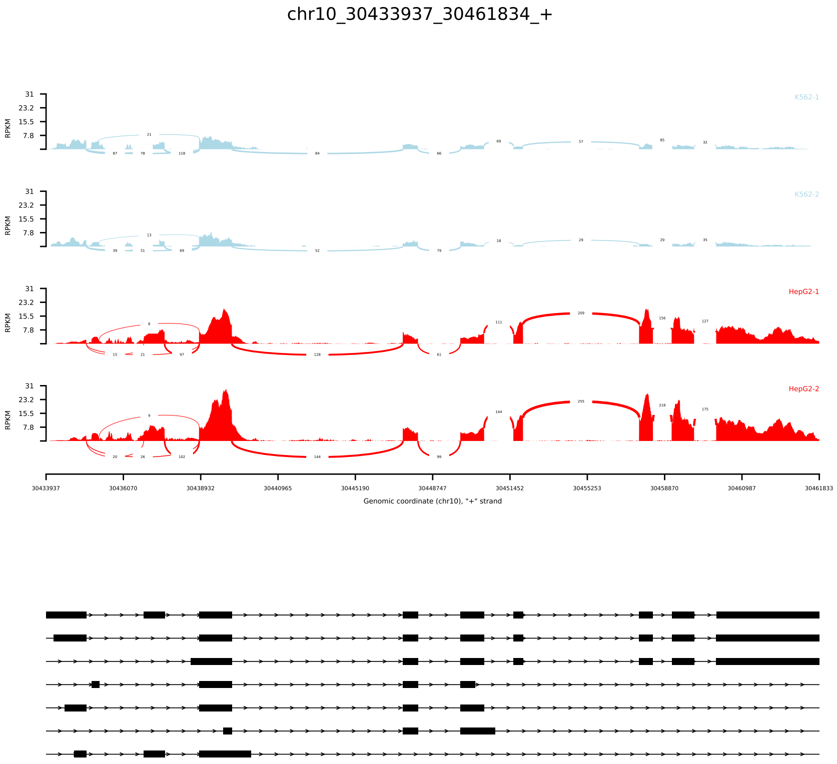

(A) MAP3K8

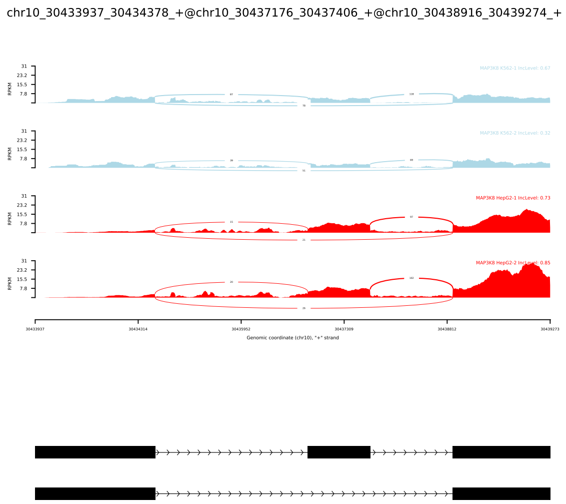

(B) Exon 2 of MAP3K8

Figure S17. (**A**) Sashimi plot showing the RNA-seq read support for the exon usage events of gene MAP3K8. (**B**) Sashimi plot showing the RNA-Seq read support for the alternative exon of MAP3K8 marked by H3K36me3 and bound by TIA1 in the cell line K562, see Figure 9B.

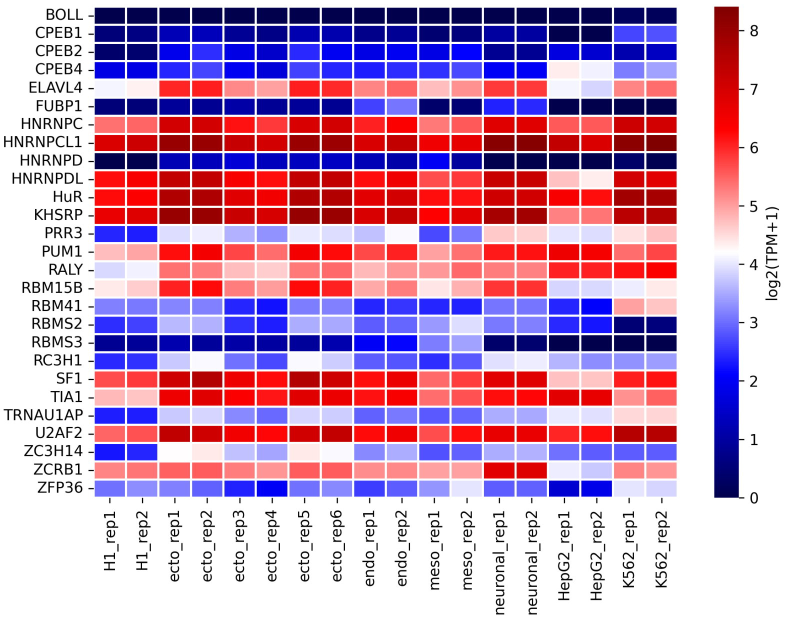

(A) Expression of Episplicing RBPs - H3K36me3

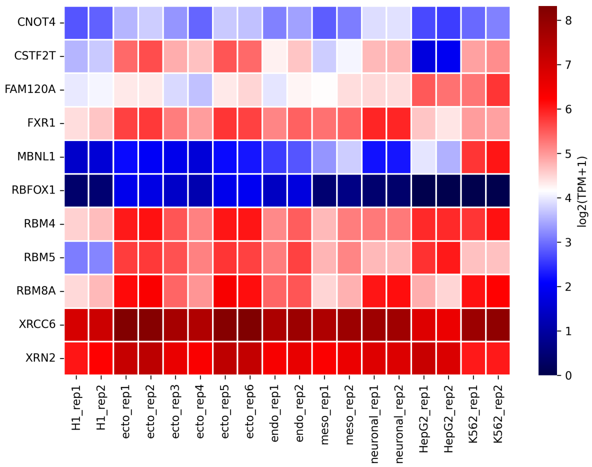

(B) Expression of Episplicing RBPs - H3K27ac

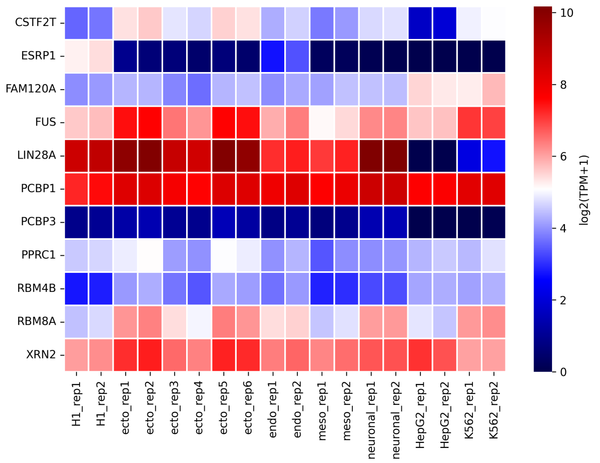

(C) Expression of Episplicing RBPs - H3K27me3

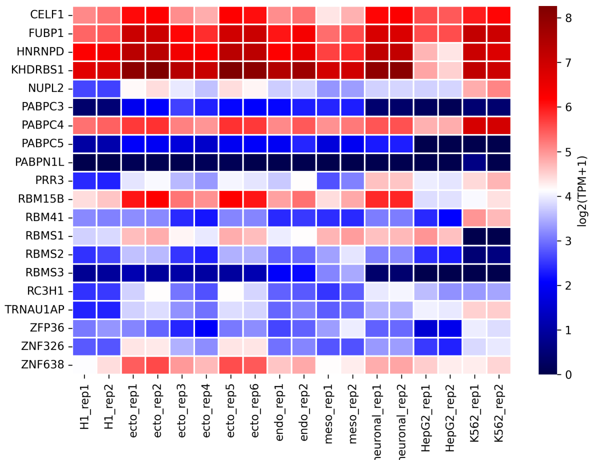

(D) Expression of Episplicing RBPs - H3K9me3

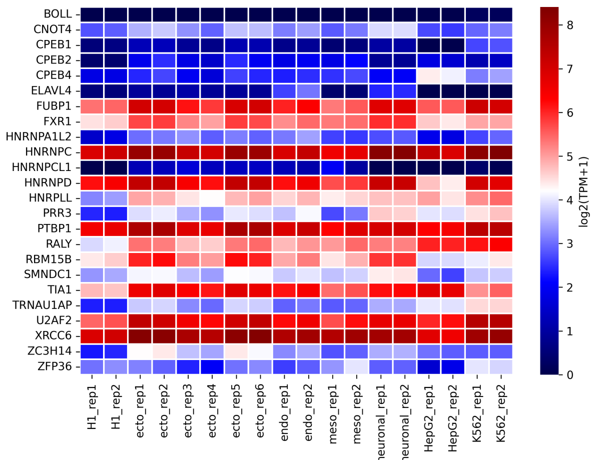

(E) Expression of Episplicing RBPs - H3K4me3

Figure S18. Expression levels of episplicing RBPs in the five embryonic and two cancer cell lines. These RBPs were predicted to bridge differential usage of exons with their local histone signal in the embryonic cell lines.
